# Infiltration of tumors is regulated by T cell-intrinsic nitric oxide synthesis

**DOI:** 10.1158/2326-6066.CIR-22-0387

**Published:** 2022-12-27

**Authors:** Pedro P. Cunha, David Bargiela, Eleanor Minogue, Lena C. M. Krause, Laura Barbieri, Carolin Brombach, Milos Gojkovic, Emilia Marklund, Sandra Pietsch, Iosifina Foskolou, Cristina M. Branco, Pedro Veliça, Randall S. Johnson

**Affiliations:** 1 Department of Physiology, Development and Neuroscience, University of Cambridge, UK; 2 Department of Cell and Molecular Biology, Karolinska Institute, Sweden; 3 Centre for Cancer Research and Cell Biology, Queen’s University, Belfast, UK

**Keywords:** Immunology, T cell, nitric oxide, antitumor immunity, tumor infiltration

## Abstract

Nitric oxide (NO) is a signaling molecule produced by NO synthases (NOS1-3) to control processes such as neurotransmission, vascular permeability, and immune function. Although myeloid cell-derived NO has been shown to suppress T-cell responses, the role of NO synthesis in T cells themselves is not well understood. Here, we showed that significant amounts of NO were synthesized in human and murine CD8^+^ T cells following activation. Tumor growth was significantly accelerated in a T cell-specific, *Nos2*-null mouse model. Genetic deletion of *Nos2* expression in murine T cells altered effector differentiation, reduced tumor infiltration, and inhibited recall responses and adoptive cell transfer function. These data show that endogenous NO production plays a critical role in T cell-mediated tumor immunity.

## Introduction

Nitric oxide (NO) is a membrane-permeable and highly labile gas with manifold effects on both cells and tissues. It has a short half-life *in vivo*, from seconds to a few minutes, and is rapidly oxidized to nitrate and nitrite (1). NO is known to play an essential role in a wide range of physiological processes, including vascular permeability, neuronal function, and immune response (2).

Mammalian tissues can reduce nitrates and nitrites to bio-active nitrogen oxides, but the majority of NO is produced by the nitric oxide synthase enzymes (NOS) (3). In mammals, there are three NOS isoforms (NOS1-3) that synthesize NO and L-citrulline from L-arginine and oxygen. NOS1 and NOS3 are calcium-sensitive enzymes, predominantly expressed in neurons and in endothelial cells, respectively, whereas the inducible NOS2 is expressed most highly in immune cells of the myeloid lineage (4). Myeloid cell-derived NO has been shown to, amongst other things, allow the resolution of inflammation through suppressing T-cell responsiveness (5–8). However, the overall significance and function of endogenous NO synthesis by T cells is still not clearly defined (9–14).

T cells cultured *in vitro* in the presence of high NO concentrations, whether derived from myeloid cells or from NO-donor drugs, have reduction in proliferative responses and cytokine release (5,15,16). However, at low doses, NO can potentiate the expansion of T-cell populations and modulate T-cell fate and metabolism (17–20). This strongly suggests that the effect of NO on T-cell function is dose-dependent. Reports using activated T cells have identified expression of NOS1 and NOS3 in human cells, whereas murine T cells predominantly express NOS2 (17,21–23). Nitric oxide production by T cells has been shown to modulate the structure of the immune synapse and to regulate T-cell differentiation (11,12,14). This finding is somewhat controversial, however, since the expression of the NOS isoforms, as well as NO production, cannot be detected in some studies of resting and activated T cells (9,10,13). In this study we investigated the role of endogenous NO production in CD8^+^ T cells and use *Nos2* deletion models specific to T cells to determine the role of NO synthesis during tumor immunity. We found that endogenous NO production by T cells has a number of unexpected roles, including the facilitation of tumor infiltration. This has implications for both the natural function of movement of activated T cells through tissues, and for the modulation of immune function by pharmacological means, including NO donors and NO synthase inhibitors.

## Materials and methods

### Study approval

Animal work was approved by the regional animal ethics Committee of Northern Stockholm, Sweden.

### Animals

C57BL/6J (CD45.2) animals were purchased from Janvier Labs. Donor TCR-transgenic OT-I mice (JAX #003831 (24)) were crossed with mice bearing the CD45.1 congenic marker (JAX #002014 (25)). The targeted deletion of *Nos2* was generated by our laboratory and created by crossing homozygous mice carrying loxP sites flanking exon 3 of *Nos2* (26) into a mouse strain of Cre-recombinase expression driven by the distal promoter of the lymphocyte-specific *Lck* gene (JAX #012837 (27)). Targeted deletion of *Hif1a* was generated by our laboratory, and achieved by crossing homozygous mice carrying loxP sites flanking exon 2 of *Hif1a* (JAX #007561(28)) into the distal promoter Lck cre transgenic strain described above. TdTomato reporter mice were generated in our laboratory by crossing homozygous mice carrying loxP-flanked STOP cassette associated to the tdTomato allele (JAX #007914 (29)) into the distal promoter Lck cre transgenic strain described above. All experiments were performed with age and sex-matched Cre-negative controls. Genotyping was performed by Transnetyx using real-time PCR. All mice used in the study were rederived from sperm into C57BL/6J recipients and used in a period of 2 years during which breeding pairs were selected to minimize inbreeding.

### Cell lines

B16-F10 cells were originally purchased from ATCC (CRL-6475) and genetically modified to express chicken ovalbumin (OVA), eGFP, and neomycin phosphotransferase (30). The resulting ovalbumin-expressing B16F10 cells were selected with 0.75 mg/mL G418 sulfate (ThermoFisher). HEK293 cells were a gift from Prof. Dantuma (Karolinska Institute, Stockholm), and MC38 cells were a gift from Dr. Asis Palazon (University of Cambridge). HUVEC were obtained from ThermoFisher (C0035C). With the exception of HUVEC, cell lines were cultured in high-glucose DMEM with pyruvate (11995065, ThermoFisher) supplemented with 10% fetal bovine serum (FBS, Sigma). HUVECs were cultured with EGM-2 Endothelial Cell Growth Medium (CC-3162, Lonza) containing EGM-2 SingleQuots Supplements (CC-4176, Lonza). All cells lines were cultured in the presence of 100 units/mL penicillin (Sigma), and 100 μg/mL streptomycin (Sigma) and in incubators with 5% CO_2_. Except for cells obtained directly from the supplier, cell lines were initially Mycoplasma tested using the MycoAlert Mycoplasma Detection Kit (LT07-118, Lonza). Cell lines were frozen at low passage number (<5) in DMEM containing 10% DMSO (Sigma) and were typically passaged 3-4 times between thawing and experimental use. Cell lines were not authenticated.

### T-cell isolation and activation

Splenic murine CD8^+^ T lymphocytes obtained from C57Bl6/J mice were purified with either positive or negative selection using microbeads (130-117-044 or 130-104-075, Miltenyi Biotec) by magnetic-activated cell sorting (MACS) and cultured in T-cell media: RPMI (21875, ThermoFisher), 55 μM β-mercaptoethanol (Sigma), 10% FBS, 100 units/mL penicillin, and 100 μg/mL streptomycin. Polyclonal mouse CD8^+^ T cells were activated with anti-mouse CD3/CD28 dynabeads (ThermoFisher) at a 1:1 cell-to-bead ratio. Purified splenic OT-I CD8^+^ T cells were activated with 0.1-1 μg/mL OVA-derived peptide SIINFEKL (ProImmune) or with anti-mouse CD3/CD28 dynabeads at a 1:1 cell-to-bead ratio. Mouse CD8^+^ T cells were expanded for up to 5 days in the presence of 10 U/mL recombinant human IL2 (Sigma). Human CD8^+^ T cells were purified from donor peripheral blood mononuclear cells (PBMCs; NHSBT or Karolinska Hospital) by positive CD8 MACS (130-045-201, Miltenyi Biotec), cultured for up to 5 days in T-cell media (without β-mercaptoethanol) supplemented with 30 U/mL recombinant human IL2 and activated with anti-human CD3/CD28 dynabeads (ThermoFisher) at a 1:1 cell-to-bead ratio.

Splenic murine naïve CD4^+^ T lymphocytes were purified from C57Bl6/J mice with negative selection using microbeads (130-104-453, Miltenyi Biotec) by MACS and cultured in T-cell media: RPMI (21875, ThermoFisher), 55 μM β-mercaptoethanol, 10% FBS, 100 units/mL penicillin, and 0.1 mg/mL streptomycin. For T_h_1 polarization, cells where cultured for 3 days with 20 ng/mL IL12 (R&D systems) and 10 μg/mL anti-mouse IL4 (BioXCell). For regulatory T-cell (Treg) polarization, cells were cultured for 3 days with 2 ng/mL TGFβ (R&D systems). For T_h_17 polarization, cells were cultured for 3 to 5 days with 2 ng/mL TGFβ, 20 ng/mL IL1β (R&D systems), 20 ng/mL IL6 (R&D systems), 10 μg/mL anti-mouse IL4, and 10 μg/mL anti-mouse IFNγ (BioXCell). CD4^+^ T cells were activated with anti-mouse CD3/CD28 dynabeads (ThermoFisher) at a 1:1 cell-to-bead ratio and cultured for 2-5 days before analysis. T cells were cultured at a density of approximately 5-10 x10^5^ cells per mL per cm^2^. T-cell purity following MACS was confirmed to be greater than 95%.

### Drug treatment of T cells

For each treatment, the same batch of cells was either incubated for 3 days with IL-2 supplemented T-cell media containing the experimental compound or DMSO vehicle control (< 0.1% of total well volume to avoid unspecific toxicity). The prolyl hydroxylase inhibitor FG-4592 (Cayman Chemicals) was used at 50 μM in wild-type mouse CD8^+^ T cells cultured in 1%O_2_. The NOS2 inhibitor 1400W dihydrochloride (Cayman Chemicals) was used at 100 μM in mouse CD8^+^ T cells transduced with VC or NOS2^OE^ vectors following enrichment of Thy-1.1^+^ cells using MACS as described below. Human CD8^+^ T cells were treated with the NO donor compound NOC-18 (Cayman Chemicals) or with the panNOS inhibitor L-NAME (Cayman Chemicals) at concentrations ranging from 1 to 256 μM. T cells treated with the different compounds were then analyzed as detailed in figure legends.

### Nitric oxide analysis

Culture media conditioned by CD8^+^ T cells for 1 day (human) or 3 days (mouse) was harvested and kept on ice. Human CD8^+^ T cells cultured for 3 days were lysed with 100 μL RIPA buffer (Thermo), and analysis of supernatants was performed following centrifugation at maximum speed for 15 minutes. Nitrate levels were quantified using a Sievers Nitric Oxide Analyzer (NOA 280i) according to the manufacturer’s instructions. Media incubated without cells or lysis buffer was used to blank for basal nitrate signals. When cell number was not the same across all conditions, results were normalized to cell counts determined by a TC20 automated cell counter (BioRad) or with the use of counting beads (C36950, ThermoFisher) followed by analysis with an Aurora flow cytometer (Cytek Biosciences).

### qRT-PCR

Total RNA was extracted from isolated CD8^+^ T cells (RNeasy kit, Qiagen), and 300 ng of RNA were used for cDNA synthesis (First-Strand Synthesis kit, Invitrogen). Quantitative real-time PCR (qRT-PCR) was performed with SYBR green (Roche) in a StepOnePlus system (Applied Biosystems). All kits were used according to the manufacturer’s instructions. Samples were run in technical duplicates. The program GeNorm (31) selected *Hprt* as the most reliable housekeeping gene to be used in the study. Primers were designed with NCBI primer blast (https://www.ncbi.nlm.nih.gov/tools/primer-blast/) and are listed in Supplementary Table S1.

### Western blotting

Cell pellets were lysed with urea-tris buffer (8 M urea, 50 mM Tris-HCl (pH=7.5), 150 mM β-mercaptoethanol), sonicated twice for 45 seconds intercalated with 1-minute incubation on ice, and then centrifuged at 14000 x *g*, 4 °C for 15 minutes. Proteins were separated by SDS-PAGE and transferred to nitrocellulose (mouse proteins) or PVDF (human proteins) membranes with a Trans-Blot Turbo Transfer System (BioRad). Membranes were incubated for 1 hours at room temperature with a blocking solution (1X ROTI Block (A151.4, Roth) used as blocking solution for mouse proteins and 5% non-fat milk in PBS + 0.1% Tween-20 was used as blocking solution for detection of human proteins). Then, membranes were incubated in 50 mL falcon tubes with a 3 mL of blocking solution containing the antibodies. Primary antibodies were used at a 1:1000 dilution and incubated overnight at 4°C in a tube roller and secondary antibodies were used at a 1:5000 dilution and incubated for 2 hours at room temperature in a tube roller. After antibody probing, membranes were washed 3 x 10 minutes in the 50 mL falcon tubes with 10 mL of blocking solution. Mouse protein extracts (25-40 μg) were probed with primary antibodies against panNOS (CST #2977), NOS2 (CST #13120) and HIF-1α (Novus NB-100-449) and detected using infrared-labeled donkey anti-rabbit (926-32213, LI-COR) and goat anti-mouse (926-68070, LI-COR) secondary antibodies in an Odyssey imaging system (LI-COR). The Revert 700 Total Protein Stain (TPS, 926-11011, LICOR) was used for the normalization of mouse protein expression. Human protein extracts (25-60 μg) were probed with antibodies against panNOS, NOS3 (CST #13120), and PPIB (CST #43603, for normalization) and detected using horseradish peroxidase-conjugated goat anti-rabbit secondary antibody (HAF008, R&D systems). Human proteins were detected with ECL Prime (GE Healthcare) and imaged with an iBrightCL1000 (ThermoFisher). M1-polarised mouse BMDM prepared as described below were used as positive control for mouse NOS2 expression whereas HUVEC cells were used as positive controls for human NOS3 expression.

### *In vitro* cytotoxicity assay

10000 B16-F10-OVA cells (target) were seeded per well in 96-well plates (flat bottom, Costar) and co-cultured for a minimum of 14 hours with 2.5 x 10^3^ to 2.5 x 10^5^ mouse CD8^+^ OT-I cells previously activated for 3 days with 1000 ng/mL SIINFEKL at 21%, 5%, or 1% O_2_. Wells were washed twice with PBS to remove T cells, and the number of remaining target cells was determined with Alamar Blue assay by culturing with 10 μg/mL resazurin (prepared from 4 mg/mL stock Sigma) and measuring the fluorescence signal (F, Ex/Em 530-560/590 nm) in a FLUOstar Omega plate reader (BMG Labtech). Cytotoxicity was calculated relative to wells with no T cells added (Pos CT) and wells with no B16 or T cells added (Neg CT): %Cytotoxicity = 100x [(F_sample_–F_Neg CT_) / (F_Pos CT_–F_Neg CT_)].

### Seahorse analysis

Mouse CD8^+^ T cells activated for 3 days in 1% O_2_ were assayed in a Seahorse Extracellular Flux Analyzer XF96 (Agilent) to determine oxygen consumption rate (OCR) and extracellular acidification rate (ECAR). The Seahorse assay was conducted in a hypoxia chamber at 3% O_2_. 1.5x10^5^ CD8^+^ T cells were plated onto poly-D-lysine-coated wells in XF RPMI medium (Agilent), pH 7.4, supplemented with 10 mM glucose (ThermoFisher) and 2 mM glutamine (ThermoFisher). Media was pre-incubated at 1% O_2_. A minimum of 5 technical replicates per biological replicate were used. During the assay, wells were sequentially injected with anti-CD3/CD28 dynabeads (4:1 bead to T-cell ratio), 1 μM oligomycin (Sigma), 1.5 μM FCCP (Sigma), and 100 nM rotenone (Sigma) + 1 μM antimycin A (Sigma).

### Transwell migration assay

Mouse CD8^+^ T cells were purified from C57BL/6j mouse spleens with CD8a positive selection microbeads (Miltenyi Biotec) and cultured for 6 days in ambient oxygen before being transferred to 1% O_2_ for 48 hours. Mouse T-cell media was supplemented with 50 U/mL recombinant human IL-2. Mouse primary lung endothelial cells (LECs) were isolated as previously described (32). LECs were cultured at 5% O_2_ in 6-well plates coated with 50 μg/mL collagen (Thermo) and cultured with EGM-2 Endothelial Cell Growth Medium containing EGM-2 SingleQuots Supplements and 100 units/mL penicillin and 100 μg/mL streptomycin. Two days before the assay, LECs were gently detached with 0.05% trypsin solution and 5-6x10^3^ cells, plated in HTS Transwell-24 units with 3.0 μm pore size (Corning) coated with 50 μg/mL, and cultured at 1% O_2_. On the day of the assay, CD8^+^ T cells were loaded for 20 minutes at 37°C in PBS with 1 μM calcein-AM solution (diluted in PBS from 1 mM stock solution prepared by adding 50 μL DMSO to 50 μg lyophilized Calcein-AM from Biolegend). After washing and aspirating LEC media on transwells, 0.2-1x10^6^ CD8^+^ T cells were added in a volume of 300 μL to the upper chamber, while the lower chamber contained 50 ng/mL murine CCL19 and murine CCL21 (R&D systems). The assay was performed at 1%O_2_. The assay media was PBS containing 2 g/L glucose (used stock solution (ThermoFisher) at 200 g/L) and 200 mg/L arginine-HCl (used 10g/L solution prepared from powdered arginine-HCl (Sigma) and pH adjusted to 7.4). The calcein fluorescent signal (Ex/Em 494/517 nm) corresponding to T cells migrating through a LEC barrier was measured 3 hours after the start of the coculture in a FLUOstar Omega Microplate Reader (BMG Labtech). Transendothelial migration was calculated relative to wells with T cells added to the bottom chamber (Max signal) and wells with no T cells added (Neg CT): %Transendothelial migration = 100x [(F_sample_–F_Neg CT_) / (F_Max signal_–F_Neg CT_)].

### Analysis of lymphocyte populations in tissues

Thymus, inguinal lymph nodes, spleen and peripheral blood were harvested from 8 to 15-weeks old female C57BL/6j *Nos2*^fl/fl^ (control) and *Nos2*^fl/fl^dlck^Cre^ animals. Thymus, spleens and lymph nodes were mashed directly against 40 μM strainers using a syringe plunger and PBS + 0.5% FBS. After spinning at 500 g for 5 minutes, cell pellets were resuspended in 1 mL ACK Lysing Buffer (A1049201, ThermoFisher) and stained for flow cytometry analysis as described below. Blood was collected from the tail vein onto heparin-treated Capillary tubes (16.443, Sarstedt) and directly stained. Blood samples were treated with BD FACS lysing solution (BD 349202) prior to flow cytometry analysis.

### Orthotopic tumor growth and tumor infiltration experiments

8 to 15-weeks old female C57BL/6j mice were inoculated subcutaneously with 5×10^5^ B16-F10-OVA or MC38 cells. For the orthotopic tumor growth experiment, tumor cells were inoculated in *Nos2*^fl/fl^ (control) and *Nos2*^fl/fl^dlck^Cre^ animals, and tumor volume was measured every 2-3 days with electronic calipers until day 30. Peripheral blood and tumor-infiltrating immune cell composition in Nos2^fl/fl^ and Nos2^fl/fl^dlck^Cre^ was analyzed 10 days after tumor inoculation. To assess infiltration of adoptively transferred OT-I CD8^+^ T cells in tumor-bearing animals, mice were conditioned for 11 days following tumor inoculation with intraperitoneal injection of 6 mg cyclophosphamide (approximately 300 mg/kg) (Sigma). On day 14, 0.5-1×10^6^ Thy-1.1-enriched transduced OT-I CD8^+^ T cells were intraperitoneally injected, and tissue infiltration was analyzed on day 19. Animals were assigned randomly to each experimental group. Tumors were processed in gentleMACS C tubes (Miltenyi Biotec). Tumors were minced in 4.5mL HBSS (ThermoFisher) using sharp dissection scissors and processed in a GentleMACS dissociator (130-093-235, Myltenyi Biotec) using the mImpTumor-02 program. After adding 0.5 mL of HBSS solution containing 20% FBS, 10 mg/mL Collagenase Type IV (17104-019, Life Technologies) and 200 U/mL DNAse I (D5025, Sigma), tumor suspensions were incubated at 37 °C for 1 hours under shaking. After further processing in a GentleMACS dissociator using the mImpTumor-03 program, tumor cell suspensions were transferred to 50 mL falcon tubes through a 40 μM strainer. Cell suspensions from thymus, spleens and liver were obtained by directly mashing the organs against 40 μM strainers using a syringe plunger and PBS + 0.5% FBS. After spinning at 500 g for 5 minutes, cell suspensions were resuspended in 1 mL ACK Lysing Buffer and stained for flow cytometry analysis as described below. As previously described, the blood was harvested from the tail vein and directly stained with fluorochrome-labeled antibodies and analyzed by flow cytometry after treatment with treated with BD FACS lysing solution.

### Adoptive cell transfer of OT-I CD8^+^ T cells

8 to 15-weeks old female C57BL/6j CD45.2^+^ mice were inoculated subcutaneously with 0.5×10^6^ B16-F10-OVA and conditioned 4 days later with a peritoneal injection of 6 mg cyclophosphamide per animal (approximately 300 mg/kg). On day 7, 0.5-1×10^6^ CD45.1^+^ OT-I CD8^+^ T cells activated for 4 days with 100 ng/mL SIINFEKL were peritoneally injected. Animals were assigned randomly to each experimental group. Tumor volume was measured every 2-3 days with electronic calipers until day 50-60. Control and experimental groups are indicated in figure legends. Peripheral blood was collected from the tail vein at days 7 and 14 days after T-cell transfer and analyzed by flow cytometry as indicated below. Tumor volume was calculated using the formula a×b×b/2 where a is the length and b is the width of the tumor.

### Generation of bone marrow-derived macrophages (BMDMs)

BMDMs were prepared from bone marrow extracted from the tibia and femur of wild-type C57BL/6J mice and cultured in non-TC-treated Petri dishes in high glucose DMEM medium containing 10% FBS, 100 units/mL penicillin, 100 μg/mL streptomycin, and supplemented with 10 ng/mL mouse GM-CSF and M-CSF (R&D Systems). After 7 days of culture, BMDMs were activated with 100 U/mL EB LPS (Invivogen) for 24 hours (M1 polarization). After washing the monolayer, cells were detached after 10 minutes incubation in CellStripper (Corning) using cell lifters. BMDMs in suspension were loaded with 100 ng/mL SIINFEKL peptide at 37 °C for 1 hour and washed before being counted and injected in 1X PBS into mice as indicated below.

### *In vivo* activation and recall experiment

OT-I T cells purified with CD8^+^ microbeads (Miltenyi Biotec) from spleens of *Nos2*^fl/fl^ (wild-type, WT; CD45.2) and *Nos2*^fl/fl^*dlck*^CRE^ (NOS2^KO^; CD45.1/CD45.2) C57BL/6j animals. WT (control) and NOS2^KO^ cells were then mixed 1:1, and a total of 2 million cells (1 million of each genotype) was injected intraperitoneally into C57BL/6J CD45.1^+^ wild-type host mice. Endogenous and adoptive populations were distinguished by the allelic variants of CD45. One day later, host mice were vaccinated intraperitoneally (i.p.) with 8x10^5^ SIINFEKL-loaded BMDMs. Peripheral blood was collected from the tail vein at days 7 and 10 after T-cell transfer and analyzed by flow cytometry following antibody staining and treatment with BD FACS lysing solution, as specified below. On day 30, animals were re-injected with 8x10^5^ SIINFEKL-loaded BMDMs, and 7 days later, the spleens, inguinal lymph nodes, and liver tissues were harvested, mashed in 40μ cell strainers into single-cell suspensions as previously described, and analyzed by flow cytometry to determine recall responses. Animals injected with PBS on day 30 were used as negative controls of the recall response. Animals were assigned randomly to each experimental group. Absolute numbers of adoptively transferred CD8^+^ OT-I cells were determined with the use of counting beads (CountBright, LifeTechnologies).

### Flow cytometry

Single-cell suspensions were stained with Near-IR Dead Cell Stain Kit (ThermoFisher), followed by surface and intracellular staining in 96-well plates with 50 μL PBS + 0.5% FBS solution containing fluorochrome-labeled antibodies (Supplementary Table S2). Staining of cytoplasmic and nuclear antigens was performed using the Fixation/Permeabilization kit (BD Biosciences) and the Transcription Factor buffer set (BD Biosciences), respectively. After each staining step, cell suspensions were washed twice with 200 μL of the buffer used to prepare antibody solutions and were centrifuged 500 g for 2 minutes. To measure IFN-γ, TNF-α, and IL-17 production, before intracellular staining, T cells were incubated in RPMI supplemented with 50 ng/mL PMA (Sigma), 1 μg/mL ionomycin (Sigma), and 5 μg/mL Brefeldin A (Sigma) for 3-4 hours. When using blood and organ-derived cell suspensions, samples were treated with Mouse Fc Block (BD Biosciences) prior to antibody staining. For proliferation assays, non-activated human CD8^+^ T cells and mouse CD8^+^ and CD4^+^ T cells isolated as shown above were loaded with CellTrace Violet (ThermoFisher) according to the manufacturer’s instructions. Samples were acquired in FACSCanto II (BD Biosciences) or in Aurora (Cytek Biosciences) flow cytometers, and data were analyzed with FlowJo version 10.

### Checkpoint blockade therapy

5x10^5^ B16-F10-OVA cells were subcutaneously implanted in Nos2^fl/fl^ (WT) and Nos2^fl/fl^dLck^Cre^(NOS2^KO^) animals. 10 days later, animals were administered 200 μg anti-PD-1 and anti-CTLA4 antibodies (InVivoMAb) or 200 μg isotype controls Rat IgG2a and Polyclonal Syrian Hamster IgG (InVivoMAb). Tumor volume was measured every 2-3 days with electronic calipers until day 45.

### Retroviral transductions

DNA encoding a codon-optimized polycistronic peptide composed of mouse Thy-1.1 and mouse NOS2 interspersed with picornavirus P2A and furin cleavage sequences were synthesized by GeneScript. The coding sequences were cloned into the gamma retroviral vector pMP71, a gift from Christopher Baum (MHH, Hannover). Addition of furin and self-cleaving picornavirus 2A sites enables post-translational separation of Thy-1.1 and NOS2, whereas the polycistronic nature of the constructs ensures equimolar production of both proteins. A plasmid encoding Thy-1.1 alone was used as vector control (VC). Protein sequences and accession numbers are available in Supplementary Table S3. The use of plasmids encoding HIF constructs and their respective sequences have been previously described (30).

For the generation of retroviral particles, sub-confluent HEK293 cultures were transfected with NOS2 overexpression vector and pCL-Eco. Helper vector pCL-Eco (for ecotropic infection) was a gift from Inder Verma (Addgene plasmid \#12371). Supernatant media containing viral particles was harvested 48 hours after transfection and used fresh or stored at -80°C. Viral supernatants were spun onto Retronectin-coated wells (Takara) at 2000 x *g* for 2 hours at 32°C and replaced with activated CD8^+^ T cells in T-cell media supplemented with 10 U/mL recombinant human IL-2, with approximately 0.5 mL viral supernatant plated with 2 x 10^5^ T cells per cm^2^. Three days after, cells were harvested, washed in a solution of PBS + 0.5% FBS and stained with Thy-1.1 (CD90.1) microbeads (130-121-273, Miltenyi Biotec) for enrichment of transduced cells with MACS following the protocol form the manufacturer.

### Statistics

Statistical analyzes were performed with Prism 9 software (GraphPad). A P value of <0.05 was considered significant, and the statistical tests used are stated in figure legends.

### Data Availability

The data generated in this study are available upon request from the corresponding author.

## Results

### T cells express nitric oxide synthases and produce nitric oxide

**Figure 1:**
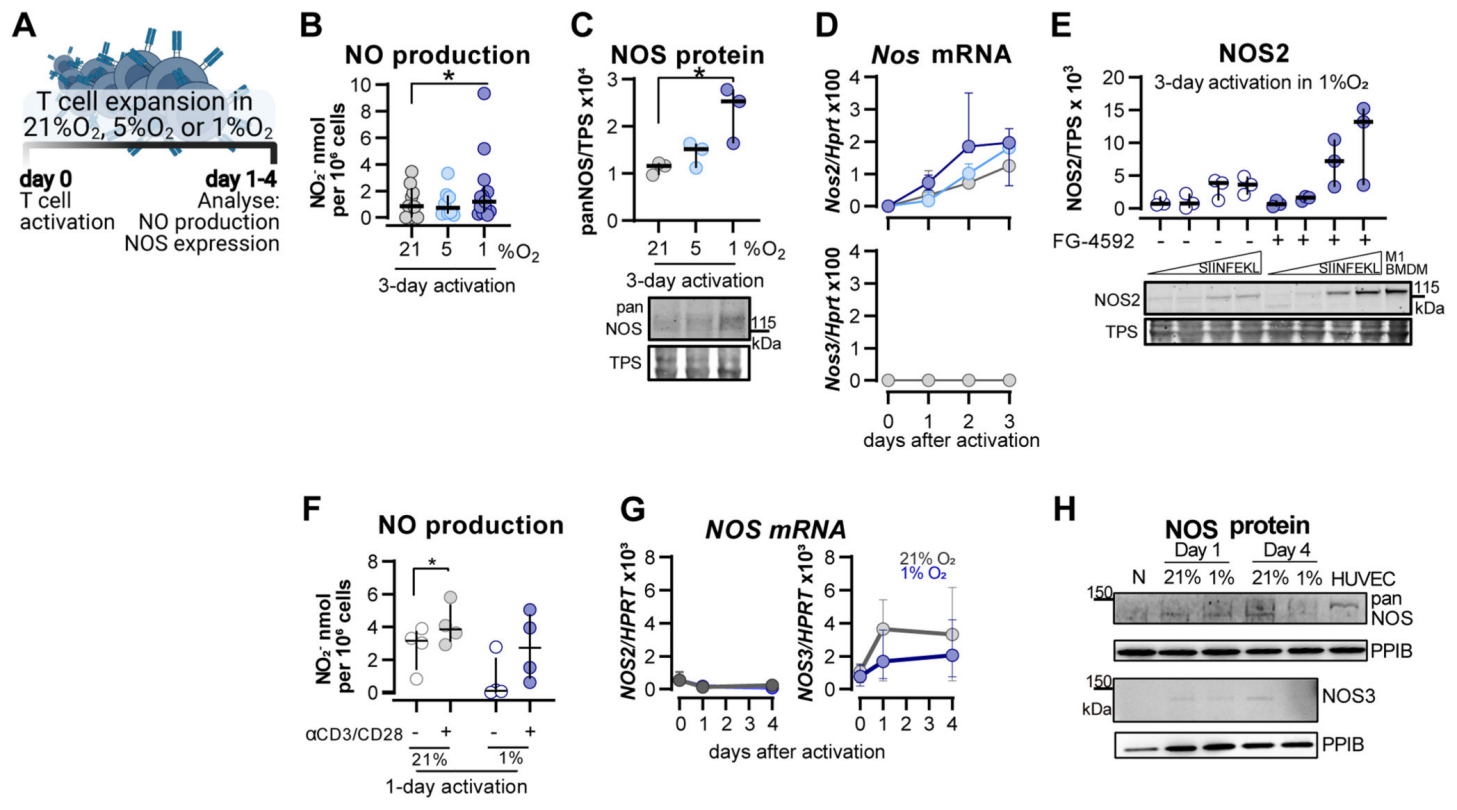
NO production and nitric oxide synthase expression in CD8^+^ T cells. **(A)** CD8^+^ T cells were activated with anti-CD3/CD28 dynabeads (or SIINFEKL peptide, when using OT-I CD8^+^ T cells) for 1-4 days in 21%, 5%, and 1% O_2_. After activation, nitric oxide (NO) production and NO synthase (NOS) expression were analyzed. **(B)** NO production determined by extracellular quantification of nitrites (NO_2_^-^, a NO byproduct) in mouse CD8^+^ T cells activated for 3 days; N=11-13. **(C)** Western blot analysis of NOS using a panNOS antibody (all isoforms detected) in lysates of mouse CD8^+^ T cell activated for 3 days. Results normalized to total protein stain (TPS, on top) and representative blot (bottom); N=3. (**D**) Time course qRT-PCR analysis of *Nos2* and *Nos3* mRNA expression in activated mouse CD8^+^ T cells; N=3-9. **(E)** Western blot analysis of NOS2 protein levels in mouse OT-I CD8^+^ T cells treated or untreated with 50 μM FG-4592 and activated for 3 days in 1% O_2_ with increasing amounts of SIINFEKL peptide (0.001, 0.1, 1 and 1000 ng/mL). Quantification normalized to TPS (top) and representative blot (bottom). Bone marrow-derived macrophages (BMDMs) polarized to M1 with 100 U/mL LPS were used as positive control for NOS2 expression; N=3. **(F)** NO production as determined by extracellular quantification of nitrites in human T cells cultured for 1 day in 21% and 1% O_2_ with or without anti-CD3/CD28 beads; N=4. **(G)** Time course qRT-PCR analysis of *NOS2* and *NOS3* mRNA levels in activated human CD8^+^ T cells. *NOS1* mRNA levels were under the detection limit; N=5-8. **(H)** Western blot analysis of panNOS (antibody detecting all NOS isoforms) and NOS3 in human CD8+ T cells. HUVEC cells were used as positive control and PPIB was used as loading control; representative of N=3. Apart from panel D and G, each datapoint represents an independent animal and results are shown as median ± interquartile range (IQR). *P<0.05: Wilcoxon matched-pairs signed-rank test.

To understand the relevance of nitric oxide (NO) in T-cell biology, we activated CD8^+^ T cells isolated from mouse spleens *in vitro* and measured the expression of NO synthases (NOS) and the production of NO during the activation process (Fig. 1A). Because NOS expression has been shown to increase with low oxygen tensions in a hypoxia-inducible factor (HIF)-dependent manner (33), we cultured cells in 21%, 5%, and 1% O_2_ for 1-3 days to assay differential NO production during activation at physiologically relevant oxygenation. Activated CD8^+^ T cells produced NO, as determined by extracellular nitrites (a byproduct of NO), with higher concentrations found in 1% O_2_ cultures (Fig. 1B, Supplementary Fig. S1A-B). Elevated NO correlated with increased NOS protein levels in T cells cultured in reduced oxygen (Fig. 1C). *Nos2* mRNA expression increased following activation of T cells, and was more robust when the T cells were cultured in lower oxygen tensions (Fig. 1D). Activation of OT-I T-cell receptor (TCR) transgenic CD8^+^ T cells with increasing doses of the cognate peptide SIINFEKL revealed a dose-dependent increase in expression of NOS2 (Fig. 1E). Enhancing HIF activity with the prolyl hydroxylase inhibitor FG-4592 further increased NOS2 protein expression (Fig. 1E), indicating that pharmacologic induction of the HIF pathway can directly augment NOS2 expression in T cells. Expression of NOS2 protein was reduced in T cells lacking HIF-1α (Supplementary Fig. S1C). Overexpression of HIF-1α, driven by various HIF constructs transduced into T cells (30), resulted in increased NOS2 protein expression; this increase correlated with increased HIF protein levels in the transduced cells (Supplementary Fig. S1D).

NO production was also detected in activated human CD8^+^ T cells and correlated with increased *Nos3* isoform mRNA and NOS3 protein levels (Fig. 1F-H, Supplementary Fig. S1E-F). These data show that NO was produced endogenously by both murine and human CD8^+^ T lymphocytes upon activation, despite the different NOS isoforms exhibited in each species.

### Pharmacological inhibition of NOS alters differentiation of human CD8+ T cells

**Figure 2:**
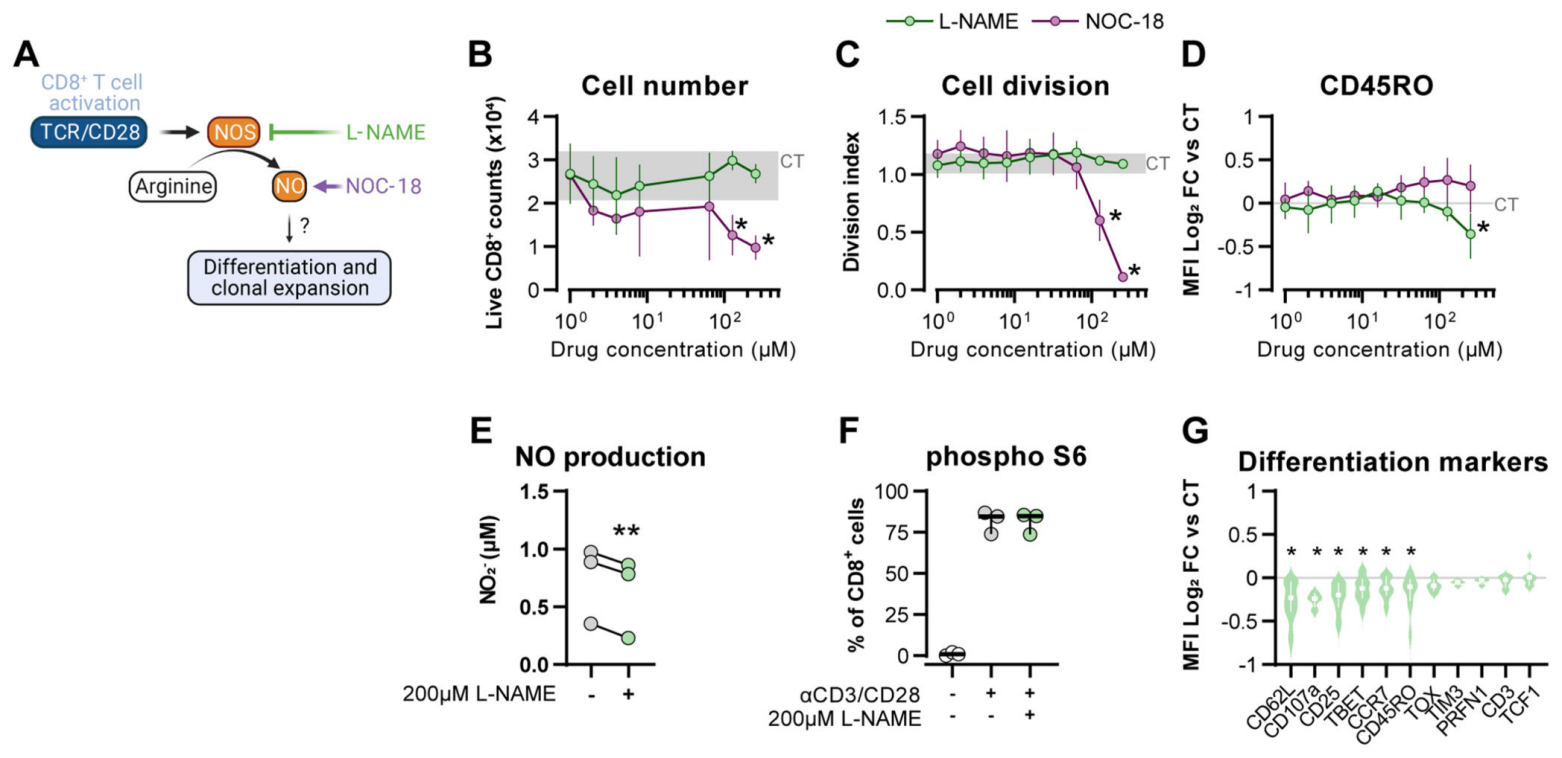
Effect of pharmacological inhibition of NO production by human CD8^+^ T cells. **(A)** Human CD8^+^ T cells were activated with anti-CD3/CD28 dynabeads in 21% O_2_ and cultured for 3 days in the presence of L-NAME (NOS inhibitor) or NOC-18 (NO donor). Flow cytometry analysis was used to assess the effects on T-cell differentiation and expansion. **(B-D)** Cell number determined with counting beads (B), cell division determined with CTV staining (C), and expression of CD45RO in cells treated with increasing concentrations of L-NAME and NOC-18. Horizontal grey line represents the DMSO control cells (CT). Cell division shown as division index and CD45RO expression shown as log2 fold-change in mean fluorescence intensity (MFI) relative to CT; n=3. **(E)** NO production determined by quantification of nitrites in lysates of human CD8^+^ T cells; N=3. **(F)** Proportion of cells positive for phospho-S6 Ribosomal Protein (Ser235/236, pS6); N=3. **(G)** Expression of differentiation markers shown as log2 fold-change in MFI relative to DMSO CT (horizontal grey line) following treatment with 200 μM L-NAME; N=4-10. Results are shown as median ± IQR. *P<0.05, *P<0.01: Wilcoxon matched-pairs signed-rank test.

To assess the role of NO in human T cells, we activated human CD8^+^ T cells in the presence of NOS inhibitor N(gamma)-nitro-L-arginine methyl ester (L-NAME) or NO donor 2,2’-(Hydroxynitrosohydrazino) bis-ethanamine (NOC-18 or DETA NONOate) (Fig. 2A). Although human CD8^+^ T cells predominantly expressed NOS3, the use of a panNOS inhibitor, such as L-NAME, ensured maximal repression of NOS activity and was possible given its reduced toxicity at high concentrations ranging to 250 μM (Fig. 2B, Supplementary Fig. S2A). Contrary to NOS inhibition, we used a NO donor compound to assess human T-cell growth and differentiation after exposure to high NO concentrations. NOC-18 was selected over other NO donors based on the slower kinetics of NO release and was detrimental to T-cell growth over the 3-day T-cell culture in the highest doses used (Fig. 2B-C, Supplementary Fig. S2A-B). L-NAME inhibited, in a dose-dependent manner, CD8^+^ T cell expression of homing markers CD45RO, CCR7, and CD62L, as well as the key transcription factor T-bet (Fig. 2D, Supplementary Fig. S2C-E). NOC-18 only significantly altered CD62L expression (Supplementary Fig. S2E).

We confirmed that treatment with L-NAME reduced endogenous production of NO in human CD8^+^ T cells (Fig. 2E). Although the downstream target of TCR stimulation, phosphorylated S6, was not altered by L-NAME treatment (Fig. 2F), several T-cell activation markers, such as the IL-2 receptor subunit CD25 and the cytotoxic marker CD107a (LAMP-1), where reduced after pharmacological inhibition of NO production (Fig. 2G). This indicated that although exogenous high levels are inhibitory for T cells, endogenous NO production can modulate T-cell activation.

### Mouse CD8^+^ T cells lacking NOS2 show altered effector differentiation and transendothelial migration capacity

**Figure 3:**
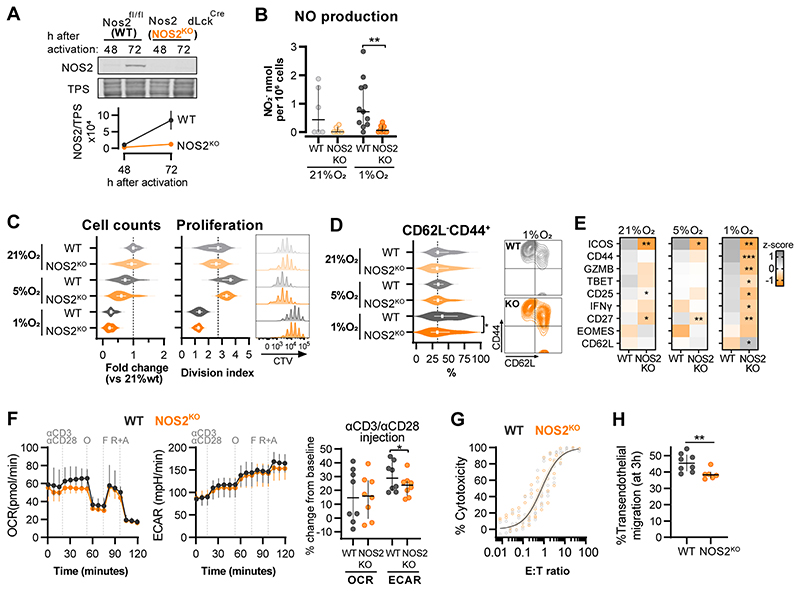
*In vitro* characterization of *Nos2* KO CD8^+^ T cells. **(A)** After activation in 1% O_2_ with anti-CD3/CD28 beads, NOS2 expression was determined by western blot in Nos2^fl/fl^ (WT, gray) and *Nos2*^fl/fl^*dLck*^Cre^ (NOS2^KO^, orange) cells. Representative blot (top) and quantification normalized to total protein stain (bottom); median ± IQR, N=2. **(B)** WT and NOS2^KO^ CD8^+^ T cells were activated for 3 days in 21% or 1% O_2_, and NO production was determined by the extracellular nitrite concentration; N=5-8. **(C)** WT and NOS2^KO^ mouse CD8^+^ T cells were activated for 72h in 21%, 5%, or 1% O_2_. Viable CD8^+^ T-cell number was determined by flow cytometry using count beads (left); cell proliferation assessed with CTV staining and expressed as division index (right); N=8-18. **(D)** Proportion of CD62L^-^CD44^+^ in cells activated as in C (left) and representative FACS plots for 1% O_2_ activated cells (right); N=11-18. **(E)** Heat map illustrating expression of markers of differentiation determined by flow cytometry in CD8^+^ T cells activated for 72h in 21%, 5%, or 1% O_2_. Increased and reduced expression of proteins are shown in gray and orange, respectively. Rows represent averaged z-scores; n=11-18. **(F)** Seahorse metabolic analysis of mouse T cells activated for 3 days in 1% O_2_, as determined by oxygen consumption rate (OCR) and extracellular acidification rate (ECAR) after injection of anti-CD3/CD28 beads or antibodies, oligomycin (O), FCCP (F), or rotenone+antimycin A (R+A) (left). Effect of T-cell activation on T-cell OCR and ECAR was determined by % change from baseline following injection of anti-CD3/CD28 beads or antibodies. Seahorse analysis was conducted in a hypoxia chamber set to 3%O_2_; N=8. **(G)** OT-I CD8^+^ T cells activated for 3 days in 1% O_2_ were co-cultured with 10000 OVA-expressing B16-F10 tumor cells at different effector:target (E:T) ratios. Cytotoxicity was assessed with Alamar blue assay after 14-18 hours of co-culture at 21% O_2_. A non-linear regression ([agonist] vs normalized response) was used to determine dose-response curves (shaded areas: 95% confidence intervals); N=4-6. **(H)** CD8^+^ T cells were activated for 6 days and incubated for 48 hours in 1% O_2_ before being loaded with calcein-AM and co-cultured with mouse endothelial cells in a transwell system. mCCL19 and mCCL21 were added to the lower chamber as chemoattractant. Calcein signal corresponding to T cells migrating through the endothelial barrier was assessed after 3 hours of co-culture in a plate reader; N=7-9. All results (median ± IQR) are pooled from a minimum of two independent experiments, and each data point from panels B, F (right), and G-H represents an independent animal: ns P>0.05, *P<0.05, **P<0.01; Mann-Whitney test relative to respective WT control.

In order to further characterize the role of NO production in T cells, we generated a mouse model with a T cell-specific deletion of *Nos2*. This was achieved by crossing homozygous mice carrying loxP sites flanking exon 3 of *Nos2* (26) into a mouse strain of Cre recombinase expression driven by the distal promoter of the lymphocyte-specific Lck (dLck) gene (27). First, CD8^+^ T cells were isolated from *Nos2^fl^*^/fl^dLck^Cre^ (NOS2^KO^) or *Nos2*^fl/fl^ (WT) control animals, activated under various oxygen tensions for 3 days, and phenotypically characterized *in vitro* (Fig. 3). Upon activation, NOS2^KO^ CD8^+^ T cells were unable to induce detectable expression of NOS2 protein (Fig. 3A) and produce NO (Fig. 3B). Despite not showing differences in expansion or proliferation following activation relative to WT control cells (Fig. 3C, Supplementary Fig. S3A), NOS2^KO^ CD8^+^ T cells cultured in 1% O_2_ showed a significantly reduced proportion of terminally differentiated cells (CD44^+^ CD62L^-^) (Fig. 3D), as well as decreased expression of markers of T-cell activation (Fig. 3E). These included CD25, CD44 (cell adhesion receptor), ICOS, and CD27 (costimulatory molecule), granzyme B and IFNγ (effector molecules), and T-bet (key transcription factor for effector T cells). We also assessed the effect of *Nos2* deletion in CD4^+^ T lymphocytes polarized in different oxygen tensions to generate T_h_1, T_h_17, and T regulatory cells (Supplementary Fig. S3B). With the exception of the increased proportion of IL-17^+^ cells in T_h_17-polarizing conditions under 1% O_2_ in NOS2^KO^ cells, no differences were found between NOS2^KO^ and WT CD4^+^ T cells in terms of polarization or expression of differentiation markers.

Reactivation of CD8^+^T cells in 1%O_2_ showed that deletion of *Nos2* reduced the increase in glycolytic rate that followed TCR triggering (as assessed by the extracellular acidification rate, Fig. 3F). *Nos2* deletion did not affect oxygen consumption rate or *in vitro* killing of tumor cells (Fig. 3F-G). Given the known role of NO in eliciting endothelium permeability, we used a coculture system to determine the degree to which T-cell infiltration through endothelial cell layers was dependent on endogenous T-cell NO production. In this experiment, T cells were inserted into a Boyden chamber above a layer of primary murine lung endothelial cells. The cytokines CCL19 and CCL21 were placed in the lower compartment as chemoattractants. Absence of *Nos2* expression significantly retarded transmigration of T cells through the endothelial cell layer (Fig. 3H).

To further understand the response of T cells to endogenous NO production, we also characterized CD8^+^ T cells overexpressing *Nos2* (NOS2^OE^). We engineered a retroviral vector encoding a polycistronic peptide composed of Thy-1.1 (surface transduction marker) and mouse NOS2 (Supplementary Fig. S3C). CD8^+^ T cell transduction with the NOS2^OE^ vector increased NOS2 protein levels in Thy1.1^+^ cells when compared to vector control (VC) or non-transduced (Thy1.1^-^) cells (Supplementary Fig. S3D). NOS2^OE^ T cells enriched with Thy-1.1 beads by MACS (Supplementary Fig. S3E) increased NO production by 30-fold (Supplementary Fig. S3F). As expected with high NO levels, expansion of CD8^+^ T cells overexpressing *Nos2* was severely impaired compared to controls, an effect that was abrogated in the presence of the NOS2 inhibitor 1400W (Supplementary Fig. S3G). In contrast to cells lacking *Nos2*, CD44 expression was increased in NOS2^OE^ T cells relative to VC; this effect was also prevented by 1400W (Supplementary Fig. S3D and S3G). *Nos2* overexpression also negatively impacted *in vitro* cytotoxicity (Supplementary Fig. S3H).

### *Nos2* deletion in T cells causes increased tumor growth

**Figure 4:**
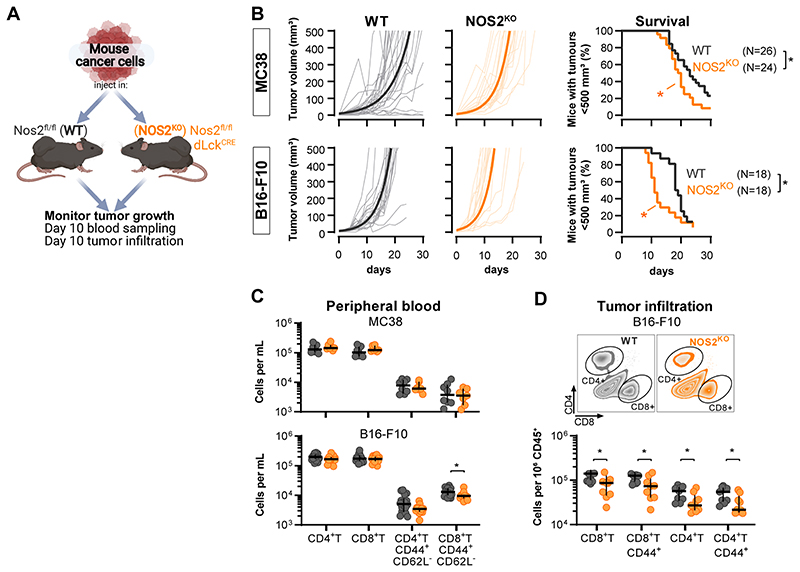
Tumor growth in animals lacking *Nos2* expression in T cells. **(A)** Tumor growth model. 5×10^5^ MC38 or B16-F10-OVA were subcutaneously injected in Nos2^fl/fl^ (WT) Nos2^fl/fl^dlck^Cre^ (NOS2^KO^) animals. On day 10 after tumor inoculation, peripheral blood and tumors were processed to single-cell suspensions and analyzed by flow cytometry. Tumor growth was monitored until day 30. **(B)** MC38 (top) and B16-F10-OVA (bottom) tumor growth data. Tumor growth curves in WT and NOS2^KO^ animals; thin lines represent individual animals and thick line represents an exponential (Malthusian) growth curve (left). Survival curves using 500 mm^3^ as threshold (right). Results pooled from two independent experiments; N=18-26 animals per group. **(C)** Immune composition was analyzed by flow cytometry on peripheral blood of animals bearing MC38 (top, N=6-8) and B16-F10-OVA (bottom, N=13-18) tumors for 10 days. Results expressed as cells per milliliter of blood; median ± IQR. **(D)** Representative flow cytometry plots from CD4^+^ and CD8^+^ T-cell infiltration in B16-F10-OVA tumors collected on day 10 following inoculation in WT and NOS2^KO^ animals (top). Immune cell infiltration in B16-F10-OVA analyzed by flow cytometry and expressed as counts per million CD45^+^ cells (bottom). Cells pre-gated on live, singlet, CD45^+^ events.; N=9, median ± IQR. Each data point represents an individual animal; ns P>0.05, *P<0.05, **P<0.05: log-rank (Mantel-Cox) test relative to WT animals (B) and Mann-Whitney test relative to WT control (C and D).

The specificity of *dLck* promoter activity in the T-cell compartment was confirmed in a Cre-loxP tdTomato reporter model (Supplementary Fig. S4A). Immune population frequencies were not altered in organs harvested from unchallenged NOS2^KO^ animals compared with (WT) littermate controls (Supplementary Fig. S4B). We directly challenged NOS2^KO^ animals with tumors to evaluate the effects of their specific deletion of *Nos2* in the entire T-cell compartment (encompassing CD8^+^ and CD4^+^ T cells) on tumor growth (Fig. 4A). NOS2^KO^ animals bearing either MC38 or B16-F10-OVA cell line-derived tumors showed increased tumor growth, and a decreased survival rate was observed in both tumor models (Fig. 4B). Blood sampling on day 10 after tumor injection showed that NOS2^KO^ animals bearing B16 tumors had a reduced proportion of terminally differentiated CD8^+^ T cells (CD62L^-^ CD44^+^) compared to WT controls (Fig. 4C). Cell suspensions obtained from B16 tumors growing for 10 days revealed that the proportion of total and CD44^+^ subsets in both CD8^+^ and CD4^+^ T cells were reduced in NOS2^KO^ animals compared to those subsets in tumors from WT control mice (Fig. 4D). The ratio between CD8^+^GZMB^+^ and CD4^+^FOXP3^+^ cells, as well as the ratios between total and CD44-expressing CD8^+^ and CD4^+^ T cells, were not altered in tumors from NOS2^KO^ animals (Supplementary Fig. S4D). The use of anti-CTLA and anti-PD1 checkpoint blockade therapy 10 days after tumor injections abrogated tumor growth differences in WT and NOS2^KO^ animals (Supplementary Fig. S4E-F). These results demonstrate the critical role played by endogenous synthesis of NO in T cells, particularly during antitumor immune responses.

### Endogenously synthesized NO facilitates CD8^+^ T-cell homing to tumors

**Figure 5:**
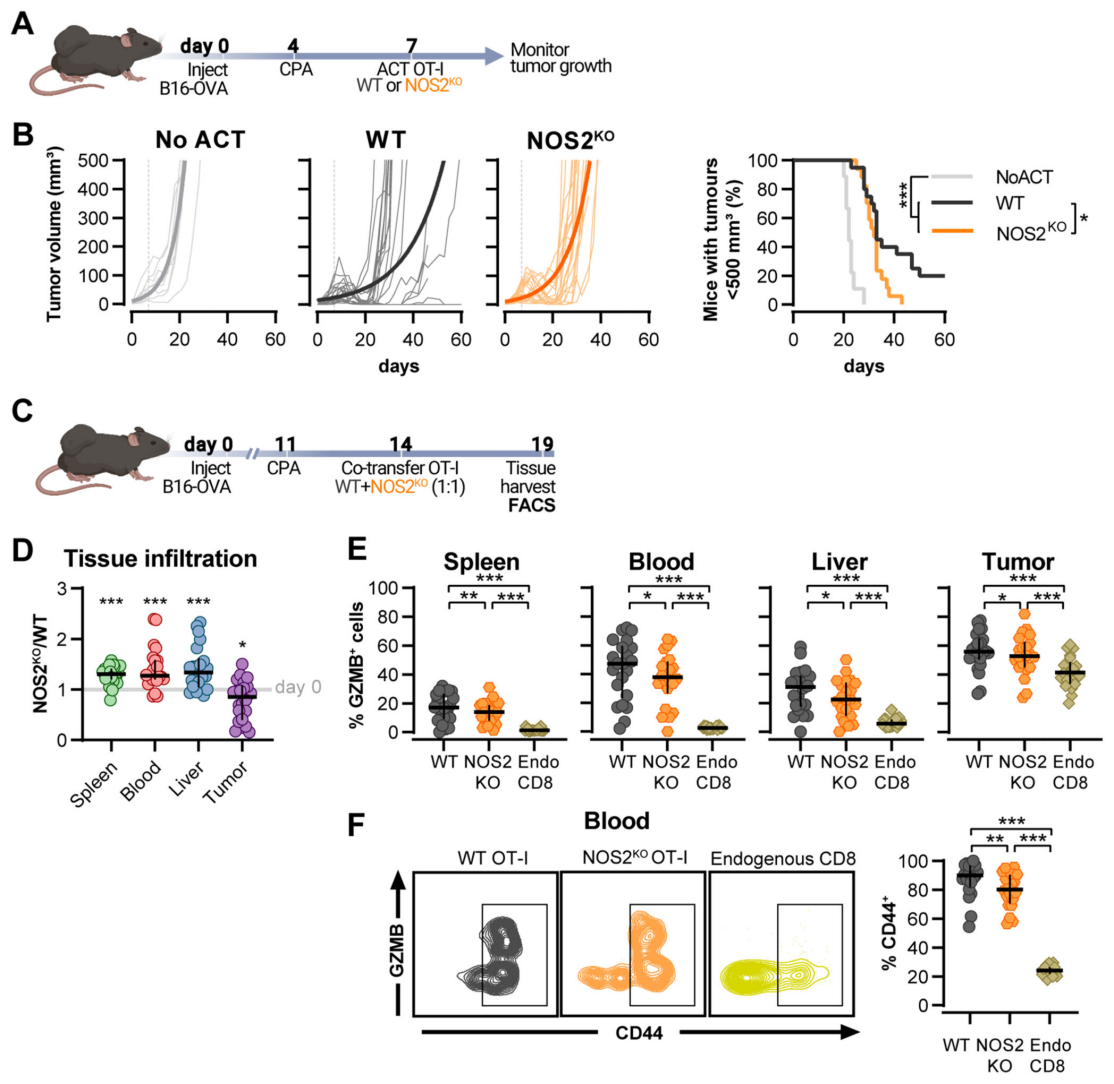
Antitumor function and tissue infiltration capacity of NOS2^KO^ OT-I T cells. **(A)** Adoptive cell therapy (ACT) model. C57BL/6j mice were injected subcutaneously with 1×10^6^ OVA-expressing B16-F10 tumor cells, and 4 days later were lymphodepleted with 300 mg/kg cyclophosphamide (CPA). Mice bearing tumors for 7 days were then intraperitoneally injected with 1×10^6^ of 4 days activated WT or NOS2^KO^ OT-I cells. Tumor growth was monitored every 2-3 days until day 60. **(B)** B16-F10-OVA tumor growth after ACT. Tumor growth curves after No ACT or ACT with VC or NOS2^KO^ OT-I cells; vertical dotted lines represent day of ACT, thin lines represent individual animals, and thick lines represent an exponential (Malthusian) growth curve (left). Survival curves using 500 mm^3^ as threshold (right); N=9-20 animals per group. **(C)** Tumor infiltration model. C57BL/6j mice were injected subcutaneously with 1×10^6^ OVA-expressing B16-F10 tumor cells, and 11 days later were lymphodepleted with CPA. Mice bearing tumors for 14 days were then intraperitoneally injected with Nos2^fl/fl^ (WT) and Nos2^fl/fl^dlck^Cre^ (NOS2^KO^) OT-I CD8^+^ T cells (1×10^6^ each in 1:1 NOS2^KO^:WT ratio). Spleen, peripheral blood, liver, and tumor were harvested on day 19 and processed to single-cell suspensions for flow cytometric analysis. Endogenous and adoptive populations were distinguished by the allelic variants of CD45. **(D)** Total OT-I T-cell expansion in all analyzed tissues expressed as a ratio between NOS2^KO^ and WT cell counts (gray horizontal line represents the NOS2^KO^/WT ratio at the time of injection); N=22, median ± IQR. **€** Percentage of cells expressing granzyme B (GZMB) within CD8^+^ T cells in all tissues analyzed by flow cytometry on day 19 (bottom); N=19-22, median ± IQR. **(F)** Representative FACS plots (left) and flow cytometry analysis (right) of percentage of CD44^+^CD8^+^ T cells in peripheral blood on day 19; N=19-22, median ± IQR. Results are pooled from at least two independent experiments, and each data point represents an independent animal; *P<0.05, **P<0.01, ***P<0.001: log-rank (Mantel-Cox) test relative to WT animals (B), One sample T-test relative to 1 (D) and Wilcoxon matched-pairs signed-rank test relative to WT control (E-F).

We next employed a mouse model of adoptive cell therapy (ACT) to assess the function of tumor specific NOS2^KO^ OT-I cells compared to WT OT-I cells (Fig. 5A). Wild-type mice were subcutaneously inoculated with B16-F10-OVA cells, lymphodepleted with cyclophosphamide (CPA), and intraperitoneally injected with 4-day activated WT or NOS2^KO^ OT-I cells. A group of animals did not receive T cells (No ACT). Tumor growth was measured for 60 days following ACT and showed that animals injected with NOS2^KO^ OT-I cells had faster tumor growth and reduced survival compared with WT controls (Fig. 5B). OT-I-receiving animals exhibited delayed tumor growth relative to No ACT controls.

We also further assessed the ability of NOS2^KO^ CD8^+^ T cells to infiltrate tumors relative to WT CD8^+^ T cells (Fig. 5C). OVA-expressing B16-F10 melanoma cells were subcutaneously injected into wild-type mice, followed by lymphodepletion with cyclophosphamide, and adoptive transfer of a cell suspension containing naïve WT and NOS2^KO^ OT-I cells at a 1:1 ratio. OT-I tissue infiltration was analyzed by flow cytometry of single-cell suspensions prepared from spleen, blood, liver, and tumors 5 days after co-transfer of T cells to tumor-bearing animals. Endogenous and adoptive populations were distinguished by the allelic variants of CD45 (Supplementary Fig. S5A). A higher proportion of NOS2^KO^ OT-I cells was found in non-malignant tissues (spleen, blood, and liver) relative to WT OT-I cells, whereas significantly fewer NOS2^KO^ OT-I cells were detected in tumors (Fig. 5D, Supplementary Fig. S5B). Deletion of *Nos2* resulted in a reduced proportion of CD8^+^T cells expressing the effector molecule granzyme B (Fig. 5E). In all tissues analyzed and relative to WT controls, NOS2^KO^ cells expressed higher levels of CD8 and CD3, which are typically downregulated after T-cell activation (Supplementary Fig. S5C). Lack of *Nos2* also decreased the proportion of tumor-specific (OT-I) CD44^+^ cells circulating in the blood (Fig. 5F).

To better understand response of T cells to endogenous NO production, we also assessed the antitumor function of OT-I cells overexpressing *Nos2* in an ACT model (Supplementary Fig. S5D). Wild-type mice were subcutaneously inoculated with B16-F10-OVA cells, lymphodepleted with cyclophosphamide (CPA), and intraperitoneally injected with 4-day activated OT-I cells transduced with retroviral vectors and purified with Thy-1.1 beads by MACS. Experimental groups included animals not receiving T cells (No ACT), animals receiving OT-I cells transduced with VC, and animals receiving OT-I cells transduced with NOS2^OE^ vectors (NOS2^OE^). Blood sampling on day 14 following ACT showed that the frequency of NOS2^OE^ cells in circulation was extremely low compared to control cells (Supplementary Fig. S5E). Tumor growth was measured for 50 days following ACT and showed that both VC and OT-I receiving animals delayed tumor growth relative to No ACT and NOS2^OE^ groups (Supplementary Fig. S5F). Tumor growth in animals receiving NOS2^OE^ OT-I cells resembled that of No ACT animals. These data showed that, similar to exposure to high NO from an exogenous source, endogenous high production of NO negatively impacted T-cell expansion and function. However, ablating endogenous production of NO reduced the ability of T cells to fully differentiate, infiltrate, and efficiently eradicate tumors.

### Decreased NO production impacts *in vivo* CD8^+^ T-cell differentiation and recall response

**Figure 6:**
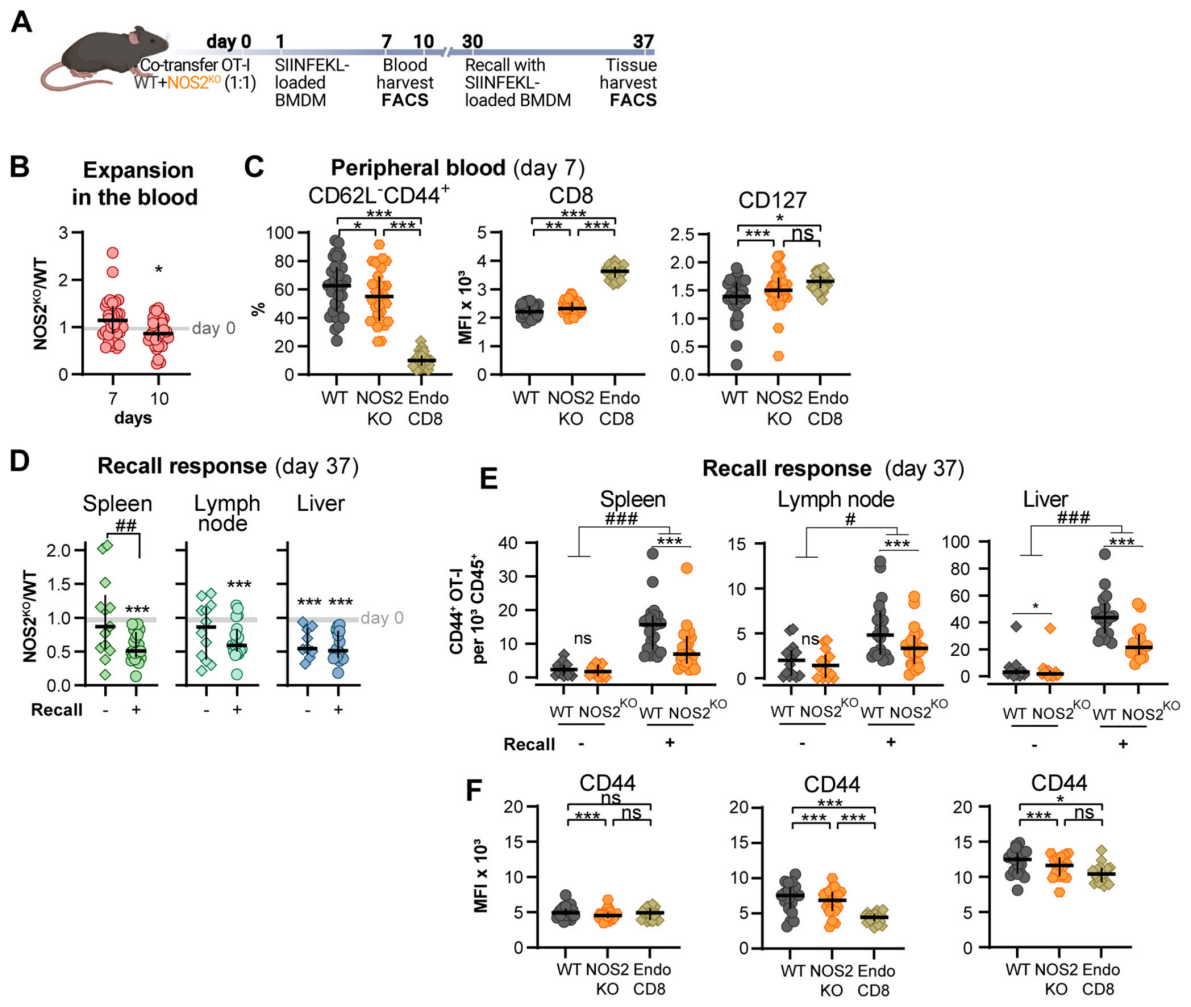
*In vivo* activation and recall response of NOS2^KO^ OT-I CD8^+^ T cells. **(A)** Scheme of *in vivo* T-cell activation and recall response model. C57BL/6j mice were injected intraperitoneally (ip) with 1×10^6^ naive Nos2^fl/fl^ (WT) and Nos2^fl/fl^dlck^Cre^ (NOS2^KO^) OT-I CD8^+^T (1:1 Nos2^KO^:WT ratio). The next day, mouse bone marrow-derived macrophages (BMDMs) differentiated for 7 days and polarized with LPS for 24h were pulsed with SIINFEKL peptide for 4 hours prior to ip injection. Peripheral blood was sampled at days 7 and 10 after BMDM transfer and analyzed by flow cytometry. SIINFEKL-pulsed BMDMs (or PBS controls) were administered again on day 30. At day 37, the spleen, inguinal lymph nodes, and a liver portion were harvested and analyzed by flow cytometry. Endogenous and adoptive populations were distinguished by the allelic variants of CD45. **(B)** OT-I T-cell expansion in peripheral blood expressed as the relative ratio between KO and WT cell counts on days 7 and 10 after BMDM injections (horizontal gray line represents the range of the initial NOS2^KO^/WT ratio); N=32-36. **(C)** Percentage of CD62L-CD44+ cells (left) and CD8 and CD127 MFI (right) of WT, KO, and endogenous CD8^+^ T cells harvested from peripheral blood on day 7; N=31 **(D)** Recall response as determined by the ratio between WT and KO OT-I CD8^+^ T-cell infiltration in the spleen, lymph node, and liver 7 days after recall with SIINFEKL-pulsed BMDMs (+) or with PBS control (-). Horizontal gray line represents the range of the initial NOS2^KO^/WT ratio; N=17-21. **(E)** Recall response as determined by amount of CD44^+^ WT and NOS2^KO^ OT-I T cells per million CD45^+^ cells infiltrated in the spleen, lymph node, and liver 7 days after recall with BMDMs or PBS control; N=9-18. **(F)** Flow cytometry analysis of CD44 in CD8^+^ T cells infiltrating the spleen, lymph node, and liver on day 37. All results (median ± IQR) are pooled from three independent experiments and each data point represents an independent animal; *P<0.05, **P<0.01, ***P<0.001: One sample T-test relative to 1 (B and D), Tukey’s multiple comparisons paired test (C-F); #P<0.05, ##P<0.01, ###P<0.001, Unpaired T-test.

We next investigated whether *in vivo* T-cell activation and recall responses, key for antitumor T cell function, were affected by the loss of *Nos2* (Fig. 6A). Naive NOS2^KO^ and WT OT-I CD8^+^ T cells were co-transferred into wild-type recipient mice in a 1:1 ratio (Supplementary Fig. S6A-B). One day later, T-cell stimulation was performed using SIINFEKL-pulsed bone marrow-derived macrophages (BMDMs). The ratio of NOS2^KO^ to WT CD8^+^ T cell counts in peripheral blood on days 7 and 10 was used to track the activation and expansion of both populations. On day 30, a recall response was triggered by injecting mice with a second dose of SIINFEKL-pulsed BMDMs, and 7 days later, the ratio of NOS2^KO^ to WT cell counts within different organs was used to determine the relative fitness of NOS2^KO^ cells during reactivation. Endogenous and adoptive populations were distinguished by the allelic variants of CD45 (Supplementary Fig. S6A). On day 7 after transfer, NOS2^KO^ OT-I cells showed a reduced proportion of terminally differentiated cells (CD44^+^CD62L^-^) and higher expression of CD8 and CD127, which is commonly associated with less differentiated T cells (Fig. 6C, Supplementary Fig. S6C). Ten days after adoptive transfer, fewer NOS2^KO^ cells were found in peripheral blood (Fig. 6B, Supplementary Fig. S6C). In the absence of an antigenic recall response, the ratio between NOS2^KO^ and WT OT-I cells was close to 1 in the spleen and lymph nodes, whereas fewer NOS2^KO^ T cells were found in the liver relative to WT cells (Fig. 6D). Recall with antigen-pulsed BMDMs led to expansion of total and CD44^+^ OT-I cells in the spleen, lymph node, and liver relative to animals receiving PBS injections instead of BMDMs (Fig. 6E, Supplementary Fig. S6D). However, NOS2^KO^ T cells were reduced in all tissues relative to co-transferred WT cells (Fig. 6E, Supplementary Fig. S6D). The spleen was the only tissue where recall responses significantly reduced the ratio of NOS2^KO^:WT compared to PBS controls (Fig. 6D), whereas only in the liver were the levels of CD44^+^ OT-I cells reduced in NOS2^KO^ cells relative to WT OT-I in the PBS controls (Fig. 6E). In all tissues analyzed after recall, NOS2^KO^ OT-I infiltrates had lower CD44 expression compared with WT controls (Fig. 6F). These data demonstrate that the endogenous synthesis of NO is an essential aspect of CD8^+^ T-cell differentiation, expansion, and recall responses *in vivo*.

## Discussion

The generation of nitric oxide in the immune system has generally focused on T cells as passive players, where myeloid or tumor cell synthesis has been shown to be a significant suppressor of T-cell activity. Although these data are compelling and borne out by our own (8) and others studies (34) of hypoxia-induced myelosuppression of CD8^+^ T-cell proliferation, it is also clear that NO can be used by different cell types to carry out different processes. In that regard, the data we present here provides evidence that NO is not solely inhibitory, as it can also significantly augment adaptive antitumor immune responses.

In this study, we generated a T cell-specific NOS2^KO^ mouse model. CD8^+^ T cells from this model showed significantly decreased synthesis of NO, which was particularly evident both after activation and in reduced oxygen settings. Previous studies of T-cell function and NOS2 have used whole animal deletion models, which are to some extent compromised by the effects of deletion of the gene in myeloid lineages (5,22). Thus, the work presented here allowed an isolated and focused approach to the importance of NOS2 expression specific to T cells.

In unchallenged animals, the immune populations in the thymus, spleen, blood, and lymph nodes of Nos2^fl/fl^dlck^Cre^ (NOS2^KO^) animals resembled those of Nos2^fl/fl^ (WT) controls. This was likely in part due to the use of a Cre recombinase promoter, which is only active in mature T cells (dLck), as the lack of NOS2 in double-positive thymocytes has been found to impair their selection (35). However, tumor growth in two different models, B16 melanoma and MC38 colon carcinoma, showed significantly increased progression and resulted in decreased survival rates when animals were implanted with T cells lacking *Nos2*. We further assessed the functional role of T cell-derived NO using tumor specific CD8^+^ OT-I cells with altered NO synthesis. Augmenting intracellular NO with *Nos2* overexpression was detrimental to the function of adoptively transferred CD8^+^ T cells. Similar to the effect of exogenous NO, derived from myeloid cells (8) or NOC-18, the rise in intracellular NO in *Nos2*-overexpressing cells suppressed T-cell expansion, which could explain the impaired tumor growth control *in vivo*. Decreasing intracellular NO synthesis with *Nos2* deletion also impaired antitumor function of cells following adoptive cell transfer. We found that this might be explained by altered CD8^+^ T-cell differentiation, recall responses, and tissue infiltration.

After TCR stimulation, either *in vitro* or *in vivo*, NOS2^KO^ CD8^+^ T cells had decreased expression of several differentiation markers (CD44, CD25, and ICOS) and higher levels of CD8 and CD127, both known to be downregulated with T-cell activation (36,37). *In vitro*, these results were only observed after culture at 1 %O_2_, keeping with the higher expression of NOS2 we and others have observed in low oxygen (33,38,39). In these same conditions, NOS2^KO^ CD8^+^ T also showed reduced expression of the effector molecules granzyme B and IFNγ, as well as changes in expression of T-bet, a key transcription factor in effector T-cell responses, thus arguing endogenous expression of NO plays a role in effector T-cell function. When compared with their WT counterparts, NOS2^KO^ CD8^+^ T cells retained the same proliferative capacity, cytotoxic function, and oxygen consumption rate, contrary to what has been shown, for example, in macrophages lacking *Nos2* (40–42). As previously shown (12,20), activation of NOS2^KO^ CD4^+^ T cells increases the proportion of T_h_17^-^expressing cells following T_h_17 polarization.

In mouse models where NOS2 mutant and wild-type T cells were co-transferred into recipient mice, the lack of NOS2 in T cells reduced their expansion in the blood and significantly reduced the recall response. The lack of NOS2 in T cells also significantly reduced transendothelial migration *in vitro* and tumor infiltration *in vivo*. Infiltration of both CD4^+^ and CD8^+^ T cells was decreased in B16-bearing Nos2^fl/fl^dLck^Cre^ animals, indicating that endogenous NO can modulate both CD8^+^ and CD4^+^ T cells. When compared with WT cells, NOS2^KO^ CD8^+^ T lymphocytes showed a reduced expression of the effector molecule granzyme B and increased expression of CD8 and CD3, which are downregulated following T-cell activation. This indicates that endogenous production of NO contributes to both to T-cell differentiation and homing of cytotoxic T cells to tumors. In fact, NOS2 expression by T cells has been shown to influence CD3^+^ cell tissue infiltration and vascular dysfunction of human allografts, which was partially reverted with 1400W treatment (43).

Increasing NO production in CD8^+^ T cells by overexpressing *Nos2* (NOS2^OE^) significantly reduced cell proliferation; this was abrogated by treatment with a NOS2 chemical inhibitor, 1400W. Although CD44 was downregulated in NOS2^KO^ T cells, it was increased in NOS2^OE^ CD8^+^ T cells, and this effect could also be reversed by 1400W. Within the population of cells transduced with the NOS2^OE^ vector, only the NOS2^+^ cells overexpressed CD44. This indicates that endogenous production of NO correlates with CD44 expression. As hyaluronic acid, the CD44 ligand, was shown to increase NO production in chondrocytes and endothelial cells, this dose-dependency of CD44 expression on endogenous NO could be explained by a positive feedback loop controlling CD44 ligand binding and cell trafficking (44,45). Given the role of CD44 in transendothelial migration of lymphocytes (46), the reduced expression of CD44 in NOS2^KO^ CD8^+^ T cells might in part explain the lower migration capacity of NOS2 mutant cells through an endothelial cell barrier, and their altered tissue infiltration pattern *in vivo* when compared to WT cells.

As seen in murine T cells, human CD8^+^ T cells produced nitric oxide, primarily through the expression of NOS3. Pharmacological inhibition of NO production with L-NAME decreased expression of the trafficking molecules CD25 and T-bet in human T cells in a manner similar to that observed in murine T cells following *Nos2* deletion. These cross-species similarities highlight the importance of endogenous NO signaling in T cells.

Our data provide evidence that NO is endogenously synthesized by T cells at low levels and has clear immunomodulatory effects on their differentiation, recall response, and tissue infiltration, with the potential to affect antitumor immunity. The data indicate that finely tuned modulation of the NO synthesis pathway might allow for improved T-cell function in immunotherapeutic settings.

## Supplementary Material

Supplementary material

Table S2

## Synopsis

Loss of nitric oxide synthase is shown to debilitate cytotoxic T cells in the therapeutic setting. Although nitric oxide is typically characterized as immunosuppressive, these data highlight its key intrinsic role in modulating T-cell tumor infiltration and suppression.

## References

[1] Wink DA, Hanbauer I, Grisham MB, Laval F, Nims RW, Laval J (1996). Chemical Biology of Nitric Oxide: Regulation and Protective and Toxic Mechanisms. Current Topics in Cellular Biology.

[2] Bogdan C (2015). Nitric oxide synthase in innate and adaptive immunity: an update. Trends in immunology.

[3] Lundberg JO, Gladwin MT, Ahluwalia A, Benjamin N, Bryan NS, Butler A (2009). Nitrate and nitrite in biology, nutrition and therapeutics. Nature Chemical Biology.

[4] Nathan C (1992). Nitric oxide as a secretory product of mammalian cells. The Faseb journal.

[5] Wei X, Charles IG, Smith A, Ure J, Feng G, Huang F (1995). Altered immune responses in mice lacking inducible nitric oxide synthase. Nature.

[6] van der Veen RC (2001). Nitric oxide and T helper cell immunity. International Immunopharmacology.

[7] Doedens AL, Stockmann C, Rubinstein MP, Liao D, Zhang N, DeNardo DG (2010). Macrophage Expression of Hypoxia-Inducible Factor-1α Suppresses T-Cell Function and Promotes Tumor Progression. Cancer Research.

[8] Gojkovic M, Cunha PP, Darmasaputra GS, Barbieri L, Rundqvist H, Veliça P (2021). Oxygen-Mediated Suppression of CD8+ T Cell Proliferation by Macrophages: Role of Pharmacological Inhibitors of HIF Degradation. Front Immunol.

[9] Thüring H, Stenger S, Gmehling D, Röllinghoff M, Bogdan C (1995). Lack of inducible nitric oxide synthase activity in T cell clones and T lymphocytes from naive and Leishmania major-infected mice. European Journal of Immunology.

[10] Bauer H, Jung T, Tsikas D, Stichtenoth D, Frölich J, Neumann C (1997). Nitric oxide inhibits the secretion of T-helper 1- and T-helper 2-associated cytokines in activated human T cells. Immunology.

[11] Ibiza S, Víctor VM, Boscá I, Ortega A, Urzainqui A, O’Connor JE (2006). Endothelial Nitric Oxide Synthase Regulates T Cell Receptor Signaling at the Immunological Synapse. Immunity.

[12] Niedbala W, Alves-Filho JC, Fukada SY, Vieira SM, Mitani A, Sonego F (2011). Regulation of type 17 helper T-cell function by nitric oxide during inflammation. Proc National Acad Sci.

[13] Geiger R, Rieckmann JC, Wolf T, Basso C, Feng Y, Fuhrer T (2016). L-Arginine Modulates T Cell Metabolism and Enhances Survival and Anti-tumor Activity. Cell.

[14] Douguet L, Cherfils-Vicini J, Bod L, Lengagne R, Gilson E, Prévost-Blondel A (2016). Nitric Oxide Synthase 2 Improves Proliferation and Glycolysis of Peripheral γδ T Cells. PLOS ONE.

[15] Marcinkiewicz J, Grabowska A, Chain B (1995). Nitric oxide up-regulates the release of inflammatory mediators by mouse macrophages. Eur J Immunol.

[16] Merryman PF, Clancy RM, He XY, Abramson SB (1993). Modulation of human t cell responses by nitric oxide and its derivative, s-nitrosoglutathione. Arthritis Rheumatism.

[17] Nagy G, Koncz A, Perl A (2003). T Cell Activation-Induced Mitochondrial Hyperpolarization Is Mediated by Ca 2+ - and Redox-Dependent Production of Nitric Oxide. J Immunol.

[18] Fiorucci S, Mencarelli A, Distrutti E, Baldoni M, del Soldato P, Morelli A (2004). Nitric Oxide Regulates Immune Cell Bioenergetic: A Mechanism to Understand Immunomodulatory Functions of Nitric Oxide-Releasing Anti-Inflammatory Drugs. J Immunol.

[19] Niedbala W, Wei X, Campbell C, Thomson D, Komai-Koma M, Liew FY (2002). Nitric oxide preferentially induces type 1 T cell differentiation by selectively up-regulating IL-12 receptor β2 expression via cGMP. Proceedings of the National Academy of Sciences.

[20] Yang J, Zhang R, Lu G, Shen Y, Peng L, Zhu C (2013). T cell–derived inducible nitric oxide synthase switches off Th17 cell differentiation. The Journal of experimental medicine.

[21] Reiling N, Kröncke R, Ulmer AJ, Gerdes J, Flad H, Hauschildt S (1996). Nitric oxide synthase: expression of the endothelial, Ca2+/calmodulin-dependent isoform in human B and T lymphocytes. Eur J Immunol.

[22] Vig M, Srivastava S, Kandpal U, Sade H, Lewis V, Sarin A (2004). Inducible nitric oxide synthase in T cells regulates T cell death and immune memory. J Clin Invest.

[23] Chen W, Li L, Brod T, Saeed O, Thabet S, Jansen T (2011). Role of Increased Guanosine Triphosphate Cyclohydrolase-1 Expression and Tetrahydrobiopterin Levels upon T Cell Activation. Journal of Biological Chemistry.

[24] Hogquist KA, Jameson SC, Heath WR, Howard JL, Bevan MJ, Carbone FR (1994). T cell receptor antagonist peptides induce positive selection. Cell.

[25] Janowska-Wieczorek A, Majka M, Kijowski J, Baj-Krzyworzeka M, Reca R, Turner AR (2001). Platelet-derived microparticles bind to hematopoietic stem/progenitor cells and enhance their engraftment. Blood.

[26] Cowburn AS, Crosby A, Macias D, Branco C, Colaço R, Southwood M (2016). HIF2α–arginase axis is essential for the development of pulmonary hypertension. Proceedings of the National Academy of Sciences.

[27] Wang Q, Strong J, Killeen N (2001). Homeostatic Competition Among T Cells Revealed by Conditional Inactivation of the Mouse Cd4 Gene. J Exp Medicine.

[28] Ryan HE, Poloni M, McNulty W, Elson D, Gassmann M, Arbeit JM (2000). Hypoxia-inducible factor-1alpha is a positive factor in solid tumor growth. Cancer Res.

[29] Madisen L, Zwingman TA, Sunkin SM, Oh SW, Zariwala HA, Gu H (2010). A robust and high-throughput Cre reporting and characterization system for the whole mouse brain. Nat Neurosci.

[30] Veliça P, Cunha PP, Vojnovic N, Foskolou IP, Bargiela D, Gojkovic M (2021). Modified Hypoxia-Inducible Factor Expression in CD8+ T Cells Increases Antitumor Efficacy. Cancer Immunol Res.

[31] Vandesompele J, Preter KD, Pattyn F, Poppe B, Roy NV, Paepe AD (2002). Accurate normalization of realtime quantitative RT-PCR data by geometric averaging of multiple internal control genes. Genome Biol.

[32] Branco-Price C, Zhang N, Schnelle M, Evans C, Katschinski DM, Liao D (2012). Endothelial Cell HIF-1α and HIF-2α Differentially Regulate Metastatic Success. Cancer Cell.

[33] Melillo G, Musso T, Sica A, Taylor LS, Cox GW, Varesio L (1995). A hypoxia-responsive element mediates a novel pathway of activation of the inducible nitric oxide synthase promoter. J Exp Medicine.

[34] Nizet V, Johnson RS (2009). Interdependence of hypoxic and innate immune responses. Nature Reviews Immunology.

[35] Cohen O, Ish-Shalom E, Kfir-Erenfeld S, Herr I, Yefenof E (2012). Nitric oxide and glucocorticoids synergize in inducing apoptosis of CD4+8+ thymocytes: implications for ‘Death by Neglect’ and T-cell function. Int Immunol.

[36] Xue H-H, Kovanen PE, Pise-Masison CA, Berg M, Radovich MF, Brady JN (2002). IL-2 negatively regulates IL-7 receptor chain expression in activated T lymphocytes. Proc National Acad Sci.

[37] Xiao Z, Mescher MF, Jameson SC (2007). Detuning CD8 T cells: down-regulation of CD8 expression, tetramer binding, and response during CTL activation. J Exp Medicine.

[38] Peyssonnaux C, Datta V, Cramer T, Doedens A, Theodorakis EA, Gallo RL (2005). HIF-1α expression regulates the bactericidal capacity of phagocytes. J Clin Invest.

[39] Takeda N, O’Dea EL, Doedens A, Kim J, Weidemann A, Stockmann C (2010). Differential activation and antagonistic function of HIF-α isoforms in macrophages are essential for NO homeostasis. Genes & Development.

[40] Lu G, Zhang R, Geng S, Peng L, Jayaraman P, Chen C (2015). Myeloid cell-derived inducible nitric oxide synthase suppresses M1 macrophage polarization. Nat Commun.

[41] Palmieri EM, Gonzalez-Cotto M, Baseler WA, Davies LC, Ghesquière B, Maio N (2020). Nitric oxide orchestrates metabolic rewiring in M1 macrophages by targeting aconitase 2 and pyruvate dehydrogenase. Nat Commun.

[42] Kobayashi S, Homma T, Fujii J (2021). Nitric oxide produced by NOS2 copes with the cytotoxic effects of superoxide in macrophages. Biochem Biophysics Reports.

[43] Koh KP, Wang Y, Yi T, Shiao SL, Lorber MI, Sessa WC (2004). T cell–mediated vascular dysfunction of human allografts results from IFN-γ dysregulation of NO synthase. J Clin Invest.

[44] Singleton PA, Bourguignon LYW (2004). CD44 interaction with ankyrin and IP3 receptor in lipid rafts promotes hyaluronan-mediated Ca2+ signaling leading to nitric oxide production and endothelial cell adhesion and proliferation. Exp Cell Res.

[45] Iacob S, Knudson CB (2006). Hyaluronan fragments activate nitric oxide synthase and the production of nitric oxide by articular chondrocytes. Int J Biochem Cell Biology.

[46] Mrass P, Kinjyo I, Ng LG, Reiner SL, Puré E, Weninger W (2008). CD44 Mediates Successful Interstitial Navigation by Killer T Cells and Enables Efficient Antitumor Immunity. Immunity.

