# Supplementary material for: Infiltration of tumors is regulated by T cell-intrinsic nitric oxide synthesis
Source: Cancer Immunol Res. Author manuscript; Available in PMC 2023 Mar 3. (PMC9975666; doi:10.1158/2326-6066.CIR-22-0387)
Supplement: Supplementary material [file EMS159149-supplement-Supplementary_material.docx]

# Supplementary Data

# Supplementary figures S1-S6, Supplementary Tables S1 and S3, and uncropped western blot images.

##### **
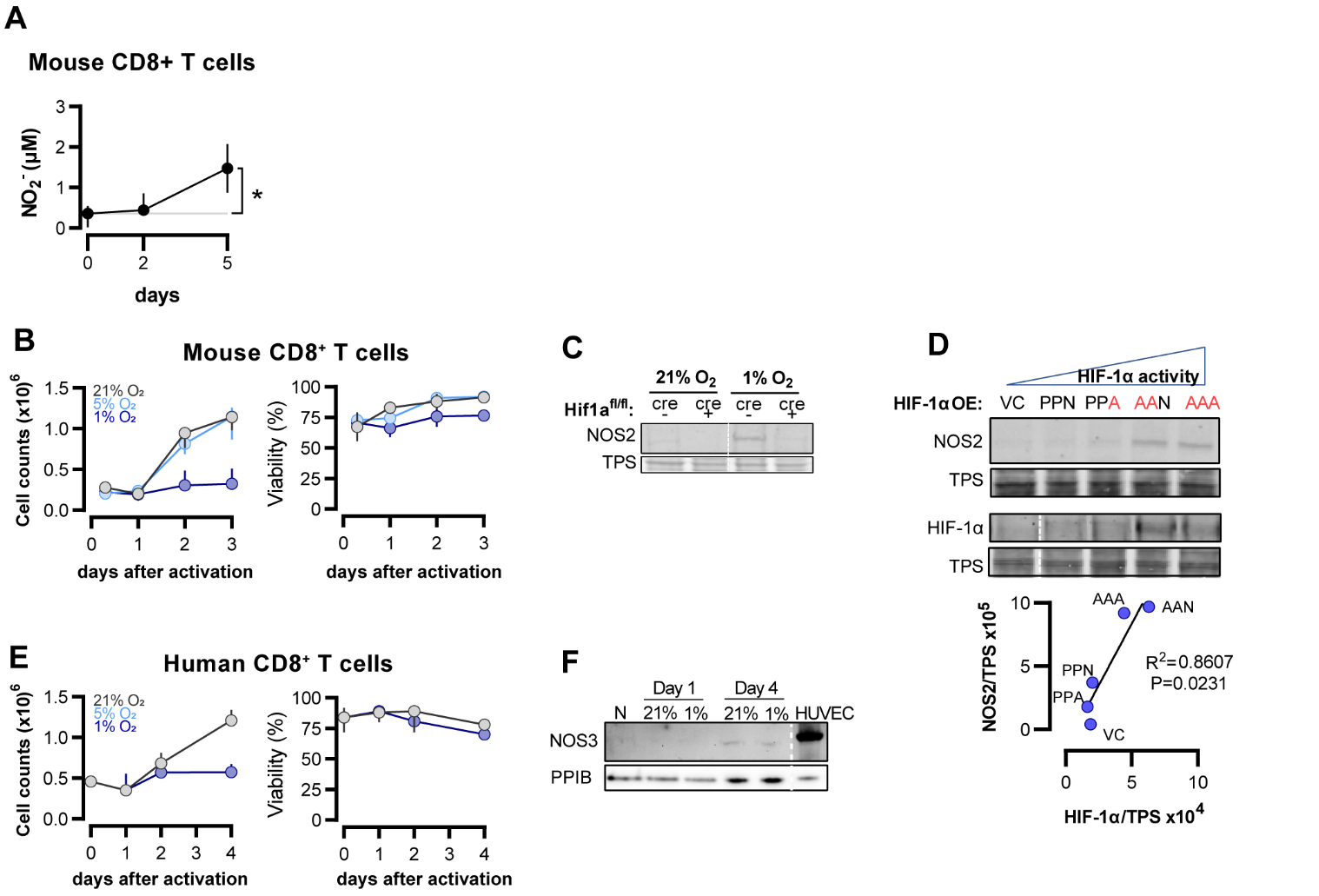
Figure S1. NO production and nitric oxide synthase expression in CD8^+^ T cells (continued from Fig. 1)**. **(A)** NO production determined by quantification of nitrites in T cell media conditioned by mouse CD8^+^ T cells before activation (day 0) or 2 and 5 days after activation with anti-CD3/CD28 dynabeads in 5% O_2_. Horizontal grey line represents the signal of cell-free media; N=3, median ± IQR. **(B)** Mouse naïve CD8^+^ T cells were activated for 3 days in 21%, 5%, or 1% O_2_ and cell counts and viability were determined with automated cell counter and Live/Dead NIR staining by flow cytometry, respectively; n=4, median ± IQR. **(C)** NOS2 protein expression in WT and HIF1 KO cells grown in 21 and 1%O_2_ for 3 days; n=1 **(D)** NOS2 expression in T cells overexpressing different HIF1a isoforms (top) and correlation between HIF-1a and NOS2 expression (bottom). VC lane on the HIF-1a blot was moved from the last lane to the first one for presentation purposes. The vertical dotted line indicates the perpendicular cut used for montage; n=1. **(E)** Human naïve CD8^+^ T cells were activated for 4 days in 21% or 1% O_2_ and cell counts and viability were determined with automated cell counter and Live/Dead NIR staining by flow cytometry, respectively; n=4, median ± IQR. **(F)** Western blot analysis of NOS3 in human CD8+ T cells using HUVEC cell as positive control and PPIB was used as loading control. For presentation purposes, blots were cut vertically between last 2 lanes as indicated by the dotted line; representative of N=3.

**
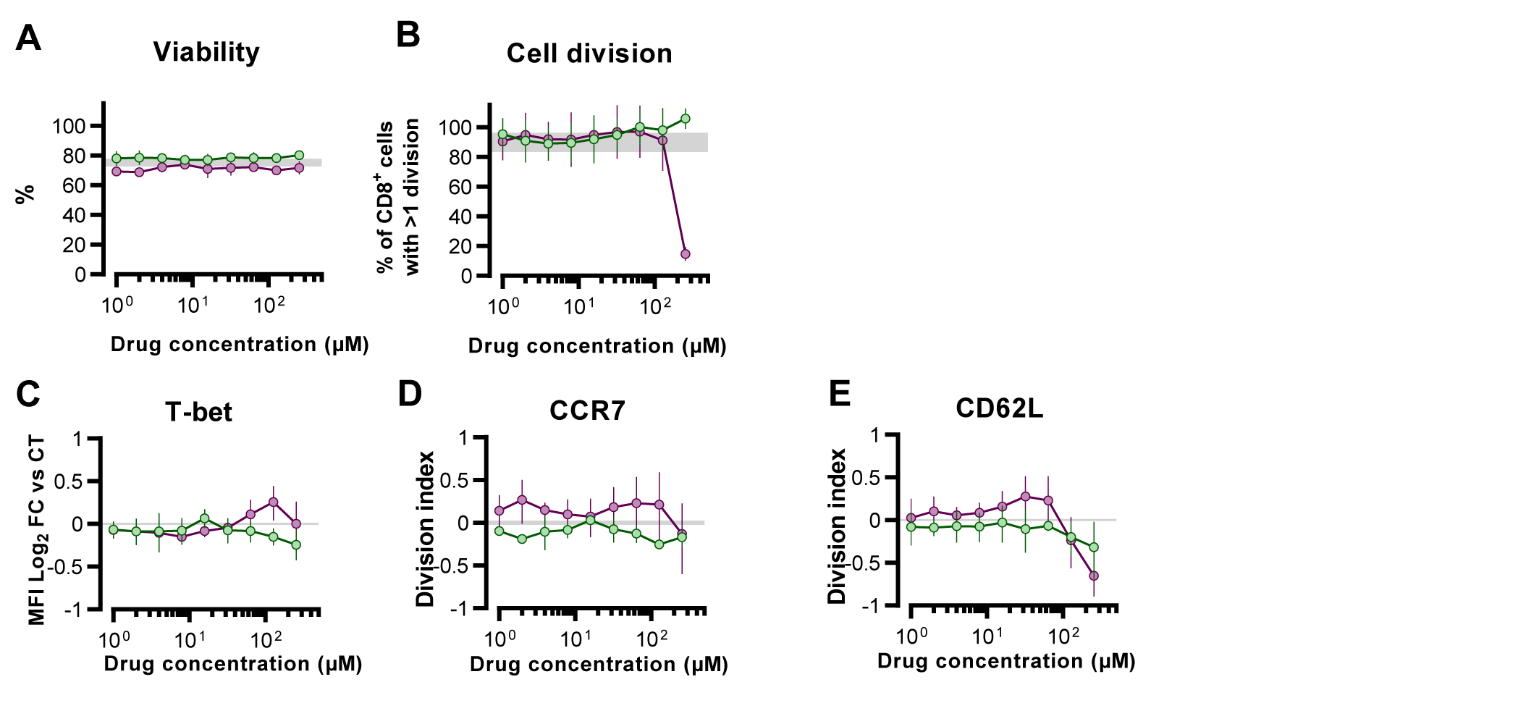
 Figure S2. Effect of pharmacological inhibition of NO production by human CD8^+^ T cells (continued from Fig. 2).** Human CD8^+^ T cells were activated with αCD3/CD28 dynabeads in 21% O_2_ and culture for 3 days in the presence of L-NAME (green) or NOC-18 (purple). Cell number determined by Live/Dead NIR staining by flow cytometry **(A)**, percentage of CD8+ T cells with more than 1 division determined with CTV staining **(B)** and expression of T-bet **(C)**, CCR7 **(D)** and CD62L **(E)**. Horizontal grey line represents the DMSO control cells (CT). Expression of differentiation markers shown as log_2_ fold change in MFI relative to CT; n=3, median ± IQR.

##### **
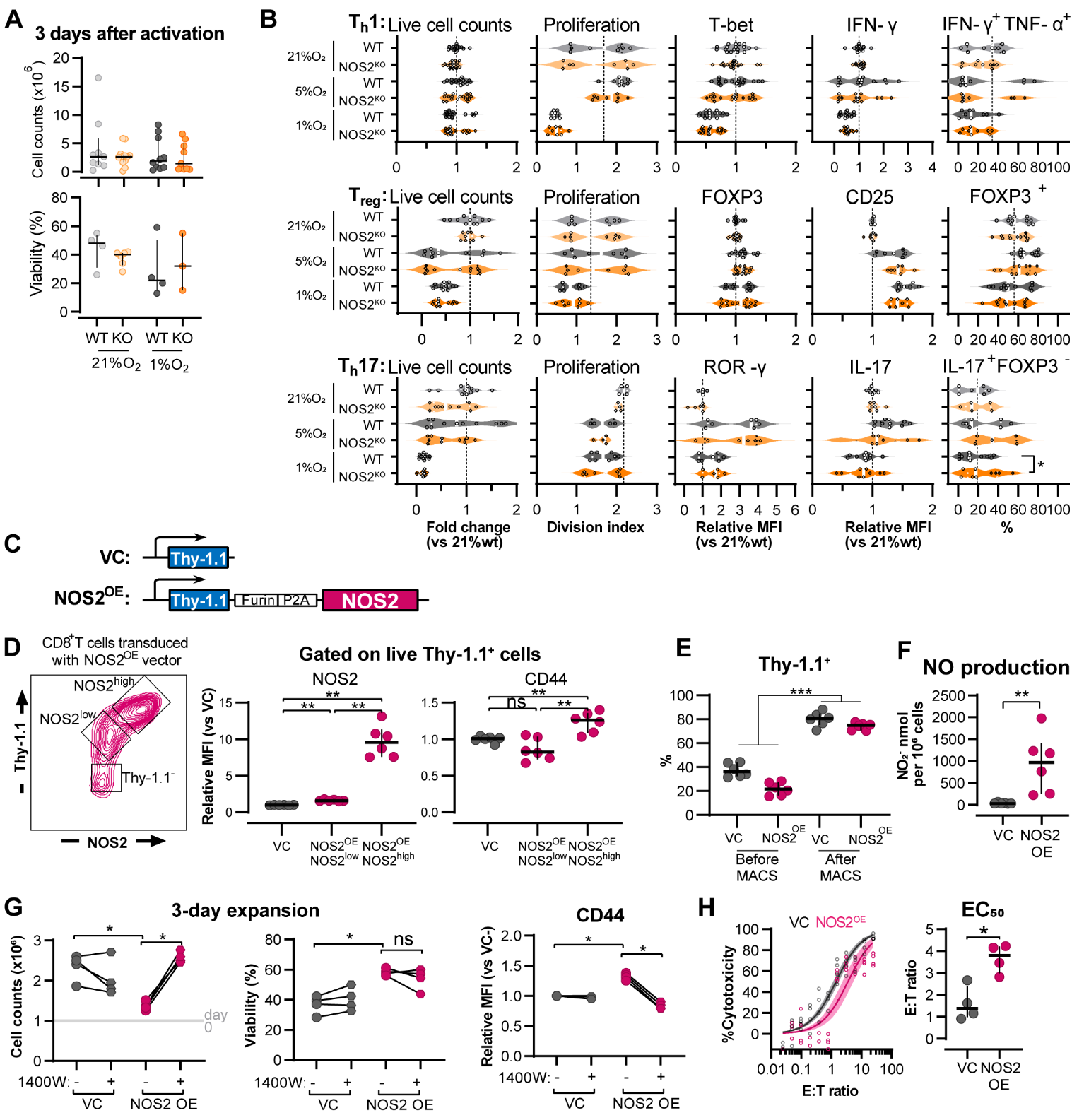
Figure S3. Effect of altered NOS2 levels in CD8^+^ T cells and CD4^+^ T cells. (A)** Mouse naïve CD8^+^ T cells were activated for 3 days in 21% or 1% O_2_ and cell counts (top) and viability (bottom) were determined with automated cell counter and Live/Dead NIR staining by flow cytometry, respectively; median ± IQR, N=5-8. **(B)** Naive mouse WT and NOS2^KO^ (KO) CD4^+^ T cells were activated for 2-5 days with αCD3/CD28 beads in different oxygen tensions and polarized with Th1, Treg and Th17 cytokines. Live counts were determined by flow cytometry using count beads and presented relative to cells grown at 21% O_2_. To assess proliferation, cells were loaded with CellTrace Violet (CTV) and the CTV dilution peaks obtained in flow cytometry were used to calculate the division index. Data on differentiation markers presented as a percentage of live CD4^+^ T cells or as median fluorescence intensity (MFI) relative to the median value in WT cells grown in 21%O_2_ from each independent experiment. Due to the bimodal distribution of fluorescent signals, MFI values of IFN-γ, FOXP3 and IL-17 were obtained from the population cells positive for IFN-γ, FOXP3 and IL-17, respectively. **(C)** Retroviral vector design for ectopic NOS2 expression (NOS2OE). After genomic integration, the retroviral long terminal repeat promoter drives the expression of a polycistronic peptide containing the surface transduction marker Thy-1.1 and NOS2, interspersed with furin-cleavage and self-cleaving picornavirus 2A sites. VC: vector control with Thy-1.1 alone. **(D)** Flow cytometry analysis of NOS2 and CD44 expression in Thy-1.1+ cells from 24 hour-activated mouse CD8+ T cells transduced with VC or NOS2^OE^ vector and expanded for 3 days; median ± IQR, N=6. **(E)** 1 day after activation, cells were transduced with VC or NOS2^OE^ vector, expanded for 3 days and enriched with Thy-1.1+ beads by magnetic-assisted cell sorting (MACS). Percentage of cells expressing Thy-1.1 before and after MACS was assessed by flow cytometry; median ± IQR, N=6. **(F)** Transduced T cells were expanded for 3 days after MACS in 21% O_2_ and extracellular quantification of nitrites was used to determine NO production; median ± IQR, N=5. **(G)** Transduced T cells were expanded for 3 days after MACS in 21% O_2_ with or without 100 µM 1400W (NOS2 inhibitor). Cell counts were determined in an automated cell counter (left) and CD44 expression was determined by flow cytometry in Thy-1.1+CD8+CD44+ cells (right); N=5. **(H)** Transduced T cells were expanded for 3 days after MACS and co-cultured with 10000 OVA-expressing B16-F10 tumor cells at different effector:target (E:T) ratios. Cytotoxicity was assessed with Alamar blue assay after 14-18 hours of co-culture. A non-linear regression ([agonist] vs normalized response) was used to determine dose-response curves (plotted with 95% confidence intervals represented in shaded areas) and EC_50_ values (plotted on the right as median ± IQR); N=4. Each data point represents an independent animal (n=8-21). ns P>0.05, * P<0.05. ** P<0.01, *** P<0.01, Mann-Whitney test relative to respective WT control.

##### **
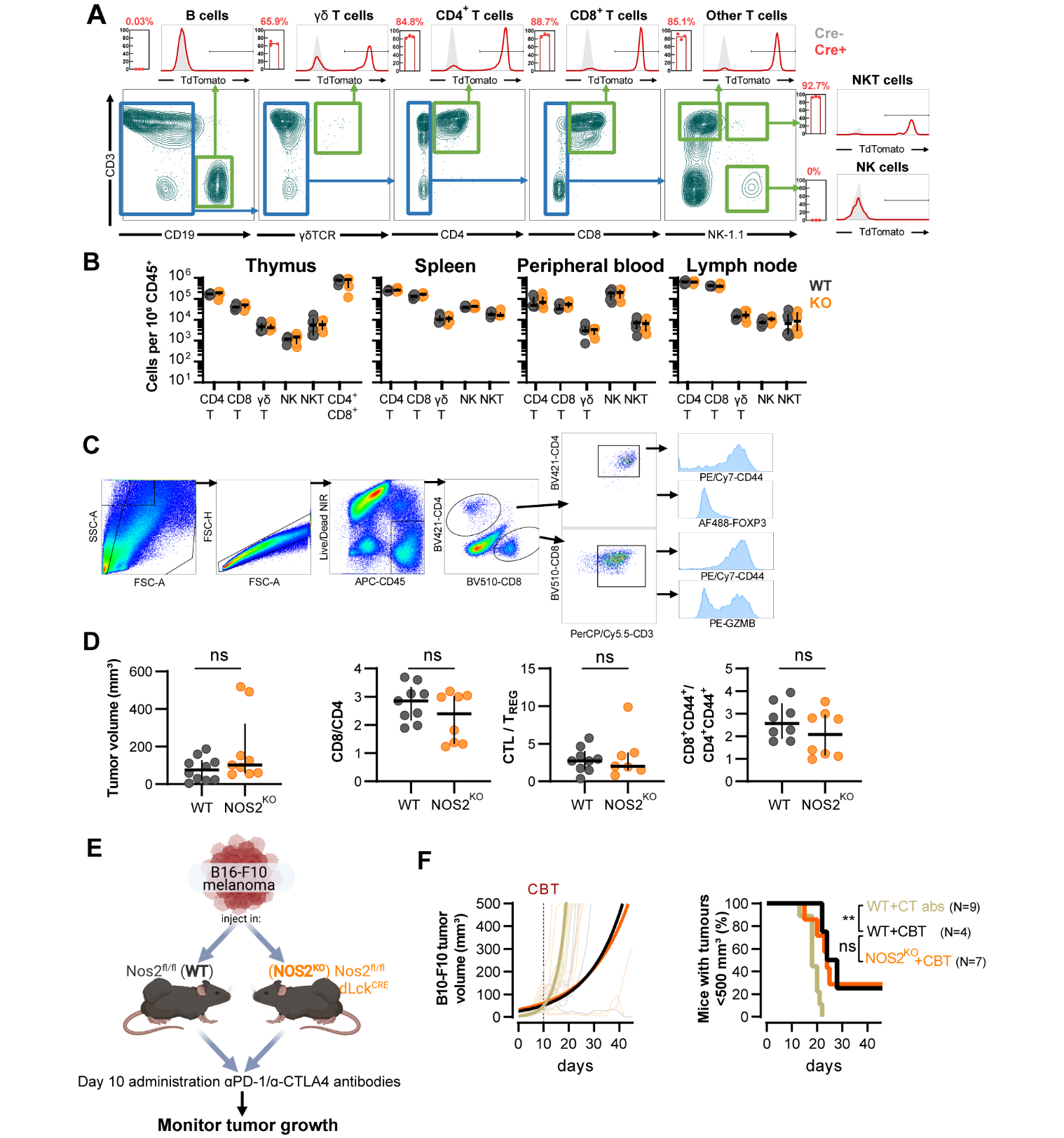
Figure S4. Characterization of the NOS2^KO^ mouse model. (A)** Expression of dlck^Cre^-driven TdTomato in different immune cells was determined by flow cytometry in lymph nodes collected from Cre+ and Cre- (CT) animals; n=3 **(B)** Thymus, spleen, peripheral blood and inguinal lymph nodes were harvested from unchallenged Nos2^fl/fl^ (WT, gray) and Nos2^fl/fl^dlck^cre^ (KO, orange) and their immune composition was determined by flow cytometry; N=6 pooled from two independent experiments. **(C)** Gating strategy relative to Fig. 4D. **(C)** Tumor volume and ratio between CD8^+^/CD4^+^, CD8^+^GZMB^+^/CD4^+^FOXP3^+^ (CTL/T_REG_) and CD8^+^CD44^+^/CD4^+^CD44^+^ cells infiltrated in B16-F10-OVA analyzed by flow cytometry on day 10 (from left to right); N=7-8, median ± IQR. **(E)** Checkpoint blockade therapy (CBT). WT and NOS2^KO^ animal bearing B16-F10-OVA tumors for 10 days were injected with 200 µg anti-PD-1 and anti-CTLA4 antibodies (WT+CBT and NOS2KO+CBT groups) and tumor growth analyzed until day 45. WT animals injected with isotype control antibodies were used as control (WT+control abs group). **(F)** ​Tumor growth curves; thin lines represent individual animals and thick line represents an exponential (Malthusian) growth curve (left). Survival curves using 500mm^3^ as threshold (bottom); N=4-9 animals per group. Each data point represents an individual animal; ns P>0.05, ** ​P<0.01: Mann-Whitney test relative to WT control (D) and log-rank (Mantel-Cox) test relative WT controls (F).

##### **
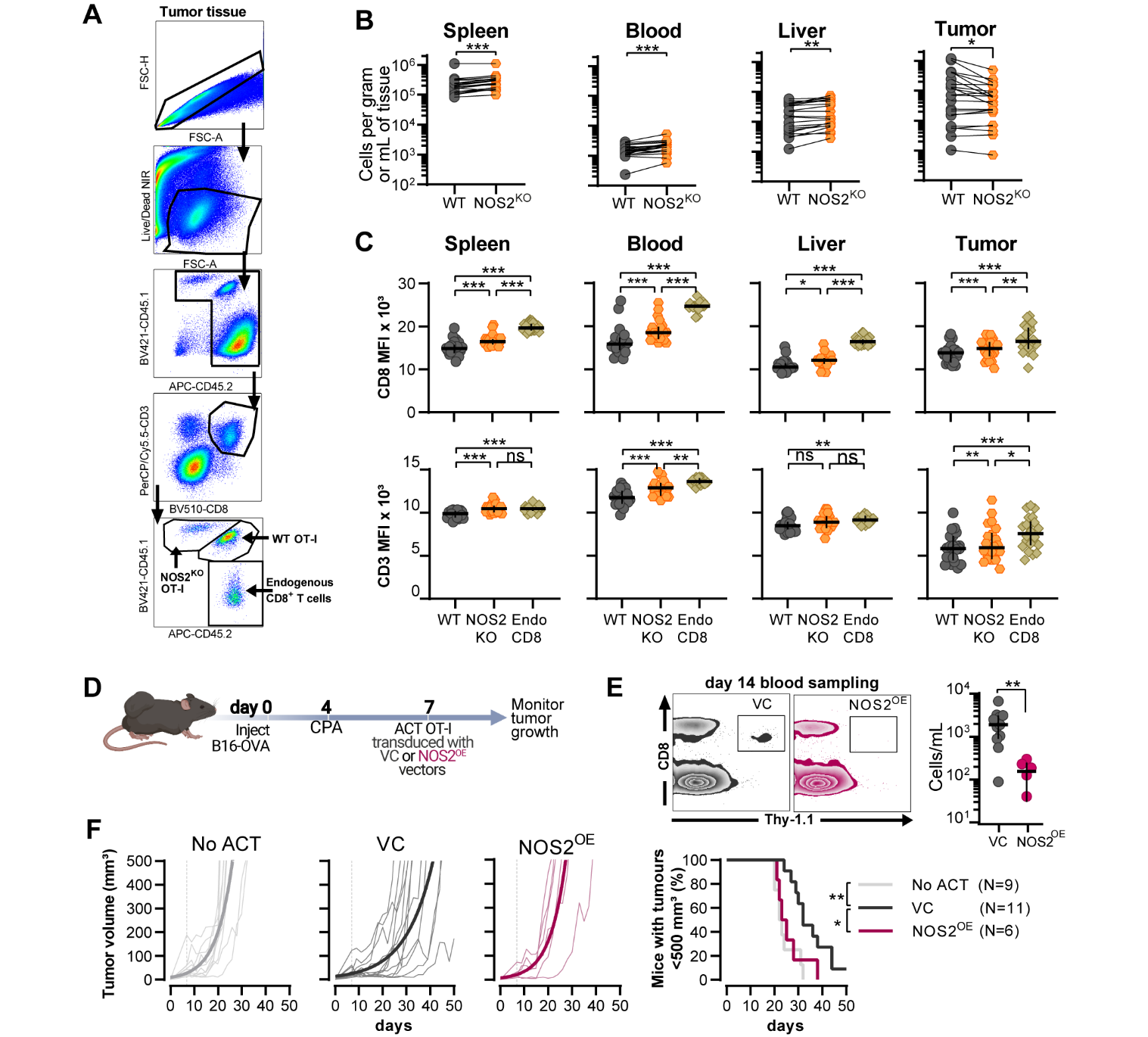
Figure S5. *In vivo* differentiation and function of T cells with altered levels of NOS2.** **(A)** Gating strategy for immune cell infiltration in tumor samples relative to Fig. 5. **(B)** OT-I cell counts per amount of tissue analyzed by flow cytometry on day 19; N=19-22, median ± IQR. **(C)** MFI of differentiation markers in CD8+ T cells infiltrated in tumors, spleen, blood and liver on day 19 analyzed by flow cytometry; N=19-22, median ± IQR. **(D)** Adoptive cell therapy (ACT) model. C57BL/6j mice were injected subcutaneously with 5×10​^5^ OVA-expressing B16-F10 tumor cells and 4 days later were lymphodepleted with 300 mg/kg cyclophosphamide (CPA). Mice bearing tumors for 7 days were then intraperitoneally injected with 5×10​^5^ of 4 days activated WT OT-I CD8^+^T cells transduced 3 days prior to ACT with VC or with NOS2^OE^ vector (NOS2^OE^ cells). Peripheral blood was sampled 14 after ACT and analyzed by flow cytometry. tumor growth was monitored every 2-3 days until day 50. **(E)** Representative flow cytometry plots (left) and frequency of adoptively transferred OT-I cells per milliliter of peripheral blood (right) analyzed 14 days after ACT. Cells pre-gated on live, singlet, CD45^+^ events; median ± IQR, N=6-11. **(F)** B16-F10-OVA tumor growth after ACT. ​Tumor growth curves after No ACT or ACT with VC or NOS2^OE^ OT-I cells; vertical dotted lines represent day of ACT, thin lines represent individual animals and thick line represents an exponential (Malthusian) growth curve (left). Survival curves using 500mm^3^ as threshold (right); N=6-11 animals per group. Each datapoint represents an independent animal; ns P>0.05, * P<0.05, ** P<0.01, *** P<0.01, Wilcoxon matched-pairs signed-rank test (B and C), Kruskal-Walis test with multiple comparisons corrected with Dunn’s test (E), log-rank (Mantel-Cox) test relative to 21% O_2_ or No t cell groups (F).

##### **
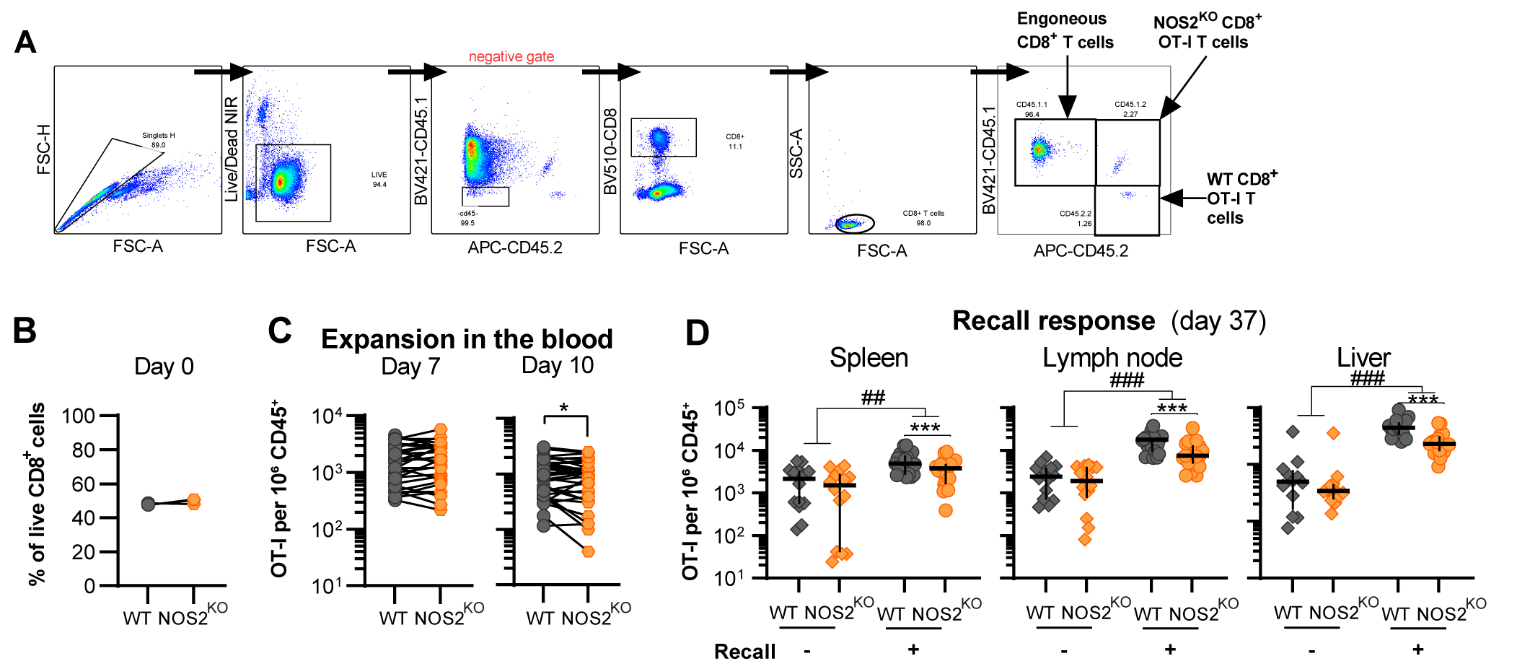
 Figure S6. *In vivo* activation and recall response of NOS2^KO^ OT-I CD8+ T cells. (A)** Gating strategy on lymph node tissue referring to Fig. 6. **(B)** Proportion of WT and NOS2^KO^ cells injected in each animal on day 0. **(C)** Expansion in the blood on days 7 and 10 of KO cells relative to WT cells; N=18-23. Results are pooled from three independent experiments and each data point represents an independent animal. **(D)** Recall response as determined by amount of total WT and NOS2^KO^ OT-I T cells per million CD45^+^ cells infiltrated in the spleen, lymph node and liver 7 days after recall with BMDM or PBS control; N=9-18, median ± IQR. * P<0.05, *** P<0.001: Tukey's multiple comparisons paired test: ## P<0.01, ### P<0.001, Unpaired T-test.

# Supplementary Tables


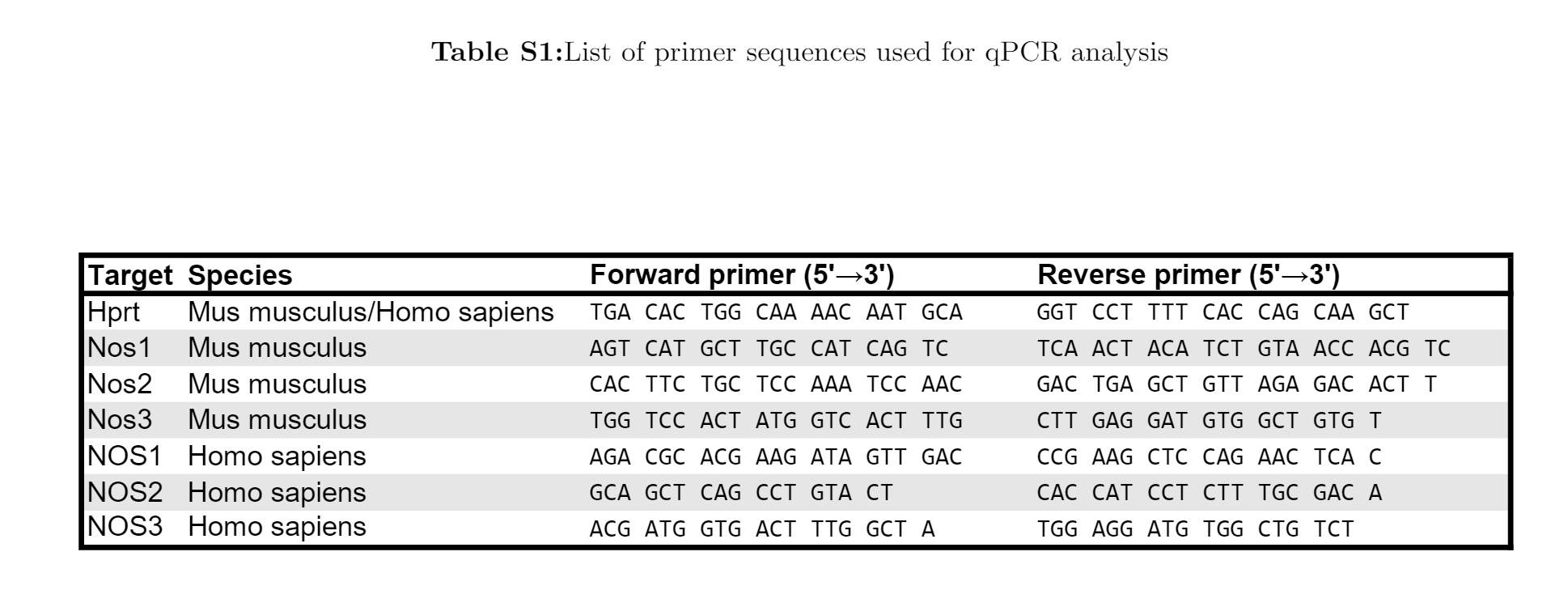


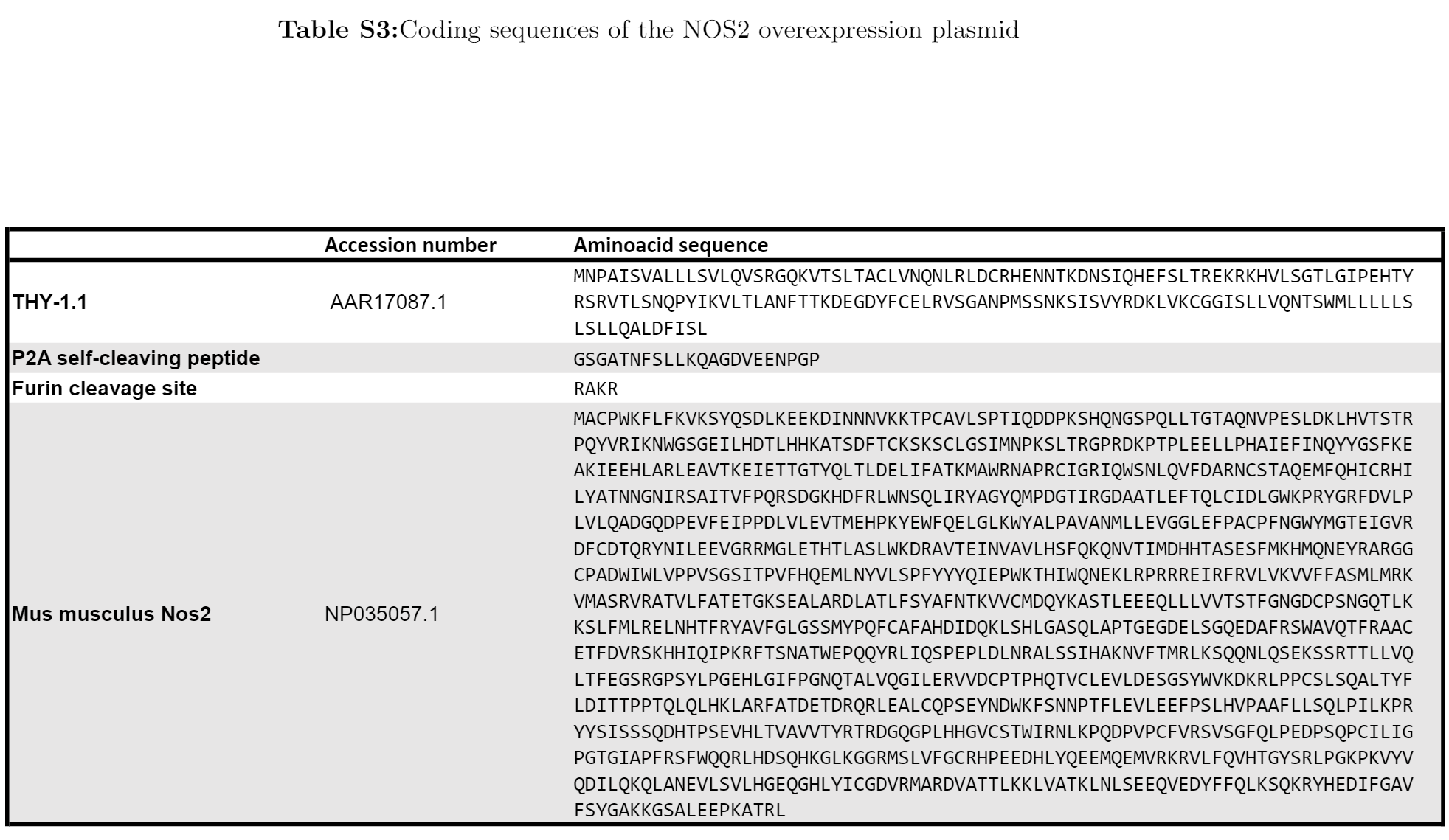


**Uncropped versions of western blots
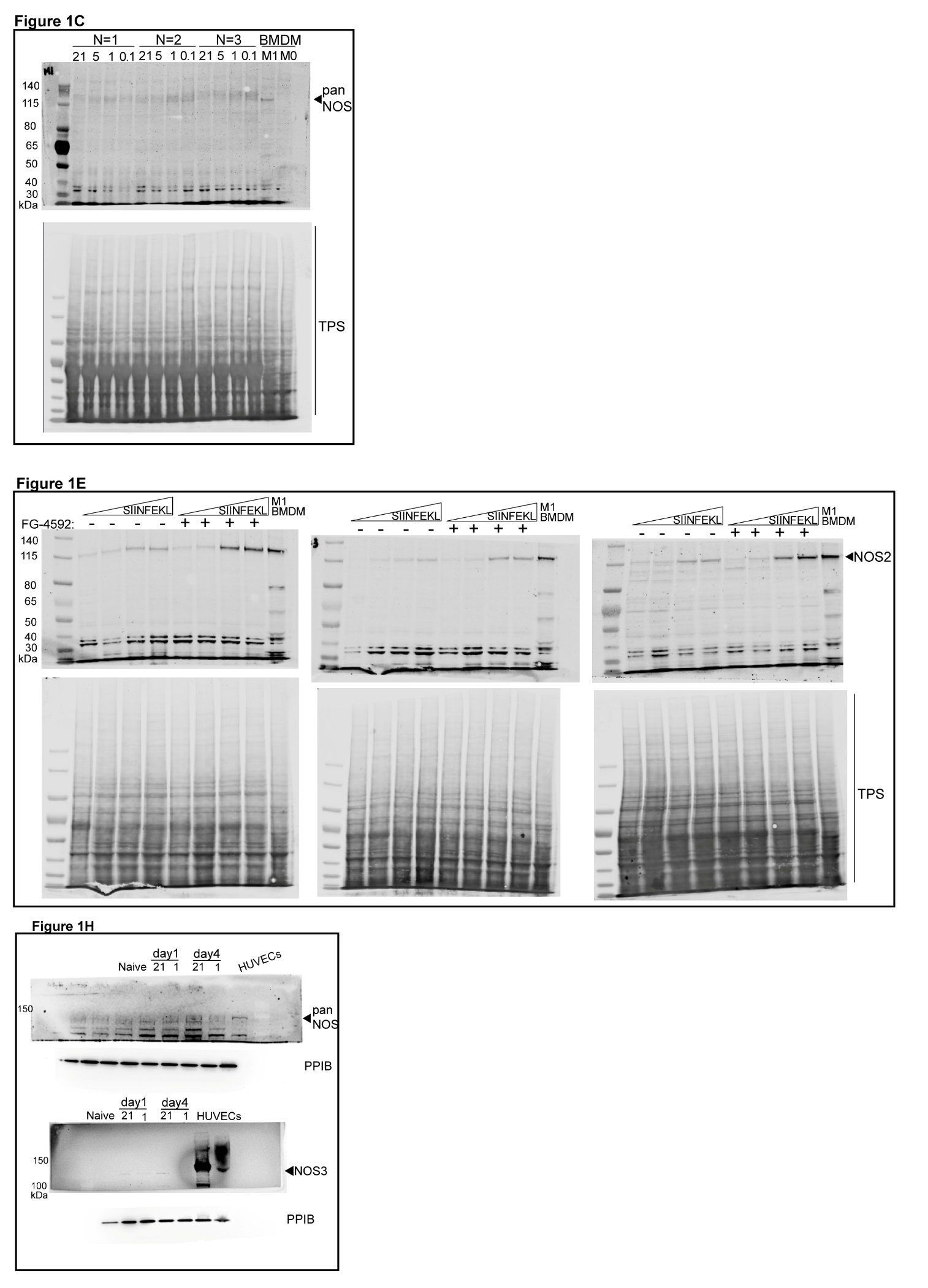

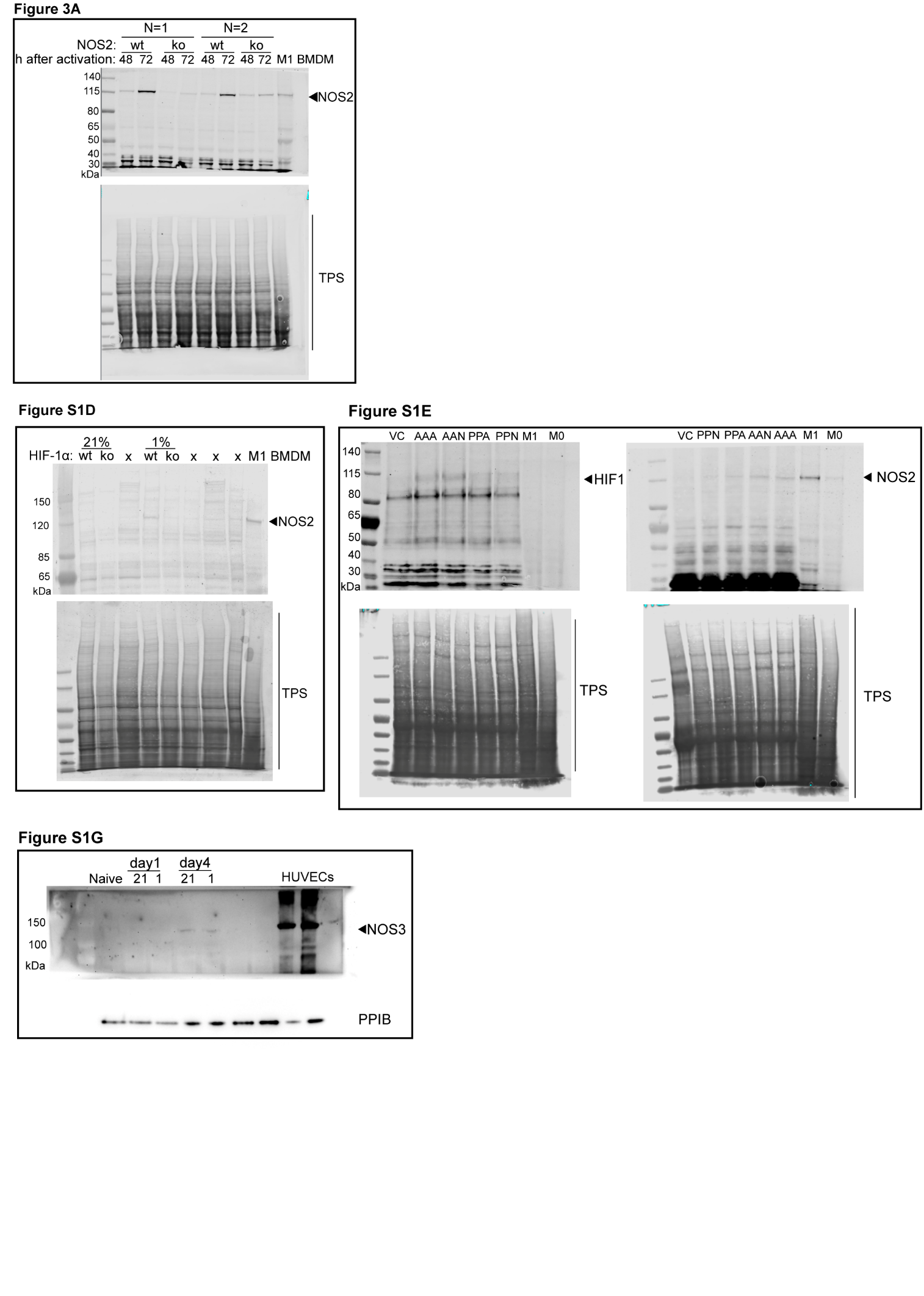
**
