# Supplementary material for: Infiltration of tumors is regulated by T cell-intrinsic nitric oxide synthesis
Source: Cancer Immunol Res. Author manuscript; Available in PMC 2023 Mar 3. (PMC9975666; doi:10.1158/2326-6066.CIR-22-0387)
Supplement: Table S2 [file EMS159149-supplement-Table_S2.pdf]

Table S2 - List of monoclonal antibodies used for flow cytometry

Mouse antigen	Fluorochrome	Clone	Supplier	Catalog #
CD3	PerCP/Cy5.5	17A2	BioLegend	100218
CD4	Brilliant Violet 421	GK1.5	BioLegend	100437
CD8a	Brilliant Violet 510	53-6.7	BioLegend	100751
CD8a	BUV395	53-6.7	BD Biosciences	565968
CD19	PE-Cy7	1D3	BD Biosciences	561739
CD25	Alexa Fluor 488	PC61.5	ThermoFisher	53-0251-80
CD27	AF700	LG.3A10	Biolegend	124239
CD44	PE-Cy7	IM7	BioLegend	103030
CD44	PerCP/Cy5.5	IM7	Biolegend	103032
CD45.1	Brilliant Violet 421	A20	BD Biosciences	563983
CD45.2	Alexa Fluor 647	104	BioLegend	109818
CD62L	Alexa Fluor 488	MEL-14	BioLegend	104420
CD62L	APC	MEL-14	Biolegend	104411
CD127	PE	A7R.34	ThermoFisher	12-1271
FOXP3	Alexa Fluor 488	150D	BioLegend	32001
FOXP3	APC	FJK-16s	ThermoFisher	17-5773-80
GZMB	PE	QA16A02	BioLegend	372207
ICOS	PerCP/Cy5.5	C398.4A	BioLegend	313518
IFN-γ	APC	XMG1.2	BioLegend	505810
IL-17a	Alexa Fluor 488	TC11-18H10	BioLegend	506910
NK-1.1	Alexa Fluor 488	PK136	BioLegend	108717
EOMES	PE	Dan11mag	ThermoFisher	1086933
NOS2	Alexa Fluor 488	CXFNT	ThermoFisher	53-5920
RORɣt	PE	Q31-378	BD Biosciences	562607
TBET	eFluor 660	eBio4B10	ThermoFisher	50-5825
TCRβ	PE/Dazzle594	H57-597	Biolegend	109239
Thy-1.1 (CD90.1)	PE-Cy7	HIS51	ThermoFisher	25-0900
TNF-α	PE-Cy7	MP6-XT22	ThermoFisher	561041
γδ TCR	APC	GL3	ThermoFisher	118115
**Human antigen**	**Fluorochrome**	**Clone**	**Supplier**	**Catalog #**
CD3	Alexa Fluor 488	HIT3a	Biolegend	300320
CD8a	BUV395	RPA-T8	BD Biosciences	563796
CD25	Brilliant Violet 510	BC96	Biolegend	302639
CD45RO	APC	UCHL1	BioLegend	304210
CD62L	PerCP/Cy5.5	DREG-56	Biolegend	304824
CD107a	Brilliant Violet 650	H4A3	Biolegend	328637
CCR7	PE/Cy7	3D12	BD Biosciences	557648
TCF1	Brilliant Violet 421	S33-966	BD Biosciences	S33-966
TBET	PerCP/Cy5.5	4B10	Biolegend	644805
TIM3	Brilliant Violet 605	F38-2E2	Biolegend	345017
PRFN1	Pacific Blue	dG9	Biolegend	308117
phospho S6	Alexa Fluor 488	D57.2.2E	Cell Signalling	4803S
